# Dendrimer-Functionalized Hybrid Materials Based on Silica as Novel Carriers of Bioactive Acids

**DOI:** 10.3390/molecules25112660

**Published:** 2020-06-08

**Authors:** Mateusz Pawlaczyk, Grzegorz Schroeder

**Affiliations:** Faculty of Chemistry, Adam Mickiewicz University in Poznań, Uniwersytetu Poznańskiego 8, 61-614 Poznań, Poland; schroede@amu.edu.pl

**Keywords:** drug delivery, hybrid materials, PAMAM dendrimers, bioactive acids, in vitro drug release

## Abstract

One of the major goals in the materials science is the design and development of non-toxic, versatile, and efficient drug delivery systems. The study reported in this paper concerns the syntheses of poly(amidoamine) (PAMAM) dendrimers with tris(2-aminoethyl)amine as an amine core and different terminal amines, and their attachment to silica matrix. The obtained ethylenediamine (EDA), triethylenetetramine (TETA), tris(2-aminoethyl)amine (TREN) and 4,7,10-trioxa-1,13-tridecanediamine (TRI-OXA) dendrimers were introduced to the support surface via an epoxy linker, leading to a loading efficiency in the range of 0.054–0.113 mmol g^−1^, determined using elemental and thermogravimetric analyses. The materials exhibited high adsorption capacities towards the chosen model drugs: folic, salicylic and nicotinic acid. The investigated adsorption processes were found to follow the Freundlich isotherm model, with indication of the drugs’ structure influence on the binding efficiency. Drug-loaded hybrid materials were also described for in vitro drug release in three pH-different paraphysiological media. The highest percentage release was obtained in the tests performed at pH 2.0, ranging between 35.42 and 99.83%. Satisfactory results and the versatility of PAMAM dendrimers may lead to the application of such materials not only as drug carriers dedicated to a wide range of pharmaceutics, but also as analytical tools for pre-concentration and/or the determination of biocompound contamination in samples.

## 1. Introduction

In recent decades, much attention has been paid to design synthesis and the final utilization of organic grafting agents dedicated to various solid supports, aiming at obtaining hybrid materials exhibiting versatile chemical and analytical applications. The common characteristic feature of such organic agents is their polyfunctionality, which affords interactions with particular analytes through non-covalent binding, such as electrostatic and hydrogen bonding, chelation, hydrophobic effect or π-π stacking of aromatic rings. Non-covalent bonds are desirable because of their high effectiveness, in particular if different types of bonds impose each other. Moreover, the low energy of non-covalent bonds permits the easy desorption of the adsorbed analytes so the materials with such bonds can be used as recyclable adsorbents and delivery systems.

One family of molecules exhibiting applicability towards diverse compounds, are dendrimers, especially poly(amidoamine) (PAMAM) dendrimers invented and introduced in 1980s by Tomalia [1]. The idea of introducing a dendritic structure as a polyfunctional domain could have originated from dendritic topology found in the natural environment, including snow crystals, neuron branches or tree roots [2]. The common features of dendrimers are: repetitiveness of structural units afforded by the repeated binding of monomers to a chosen core (branching center), multifunctionality gained with the introduction of peripheral functional groups and the presence of internal cavities as a series of gaps between the synthesized branches. More precisely, PAMAM dendrimers consist of an amine core (ammonia or polyamine, e.g., ethylenediamine, diethylenetriamine, etc.), which makes the starting point for building amidoamine branches and undergoes sequential expansion. Each step of introducing the next peripheral amino component leads to the layered expansion of the molecule called dendrimer generation (G*x*, where x is the generation number). As the dendrimer generation increases, the value of the stability constant of the dendrimer-analyte complex increases. What is more, multiple free terminal amino groups on the dendrimers’ ultimate layer allow their further functionalization with several organic domains, which may tune or introduce new interacting sources, such as: polyethylene glycol (PEG), acetyl, long-chain fatty acids, amino acids or targeting domains [3]. Therefore, PAMAM dendrimers and their derivatives may find attractive biological applications either in the native form or immobilized on a support surface. These two types of species of dendrimers and their derivatives can be subjected to in vitro and in vivo studies, in order to assess their desirable biological effectiveness and also cytotoxicity, permeability or degradability.

The drug delivery potential of bare PAMAM dendrimers is strictly related to their multifunctional structure. Namely, multiple amine and amide groups on the surface or in the dendrimer’s interior allow the non-covalent electrostatic or hydrogen bonding between the dendrimers and drug molecules [4]. Moreover, the ‘internal architecture’ of PAMAM dendrimers allows the effective physical entrapment of drugs in dendritic internal cavities, whose size is dependent on the size of the core molecule and branching units, determining the distance between the subsequent branches. The aforementioned structural features of dendrimers permit the adsorption of drug molecules to the dendrimer’s surface or permit their diffusion into the dendrimer’s interior, leading to host-guest supramolecular complexes. Although PAMAM-drug conjugates may be used as therapeutics targeted delivery systems with an enhanced transmembrane permeability which may dissolute already in blood plasma, the increase in the dendrimer generation leads to its higher cytotoxic effect towards cells. It is strongly connected with the interaction between the polycationic macromolecule and the negatively charged glycocalices, which are one of the major cell membrane constituents, triggering apoptotic cell death [5,6,7,8,9]. Densely charged PAMAM dendrimers of generation 7.0 have been also proved to enhance the aggregation of several proteins in the bloodstream, causing their deactivation [8]. The prevention of negative interactions of the dendrimers with proteins, membrane constituents, etc. is therefore connected with a decrease in the surface amine groups reactivity by their conjugation with e.g., PEG, lipophilic carboxylic acid with short hydrocarbon chains, surfactants, acetyl groups, amino acids (arginine and ornithine), biotin or dimethyl itaconate [10,11,12,13,14,15,16,17], as well as with folic acid or octeroide (somatostatin analogue) as targeting domains [18,19].

Another possibility of shielding the polycationic character of PAMAM dendrimers is their immobilization on support, leading to the group of novel dendrimer-functionalized hybrid materials. Such hybrids exhibit physicochemical features common for native dendrimers and platforms, but also gain the character of a functionalized material-finding application as sorbents: molecular scavengers, materials for analytes extraction, pre-concentration systems and delivery systems of biomolecules or nucleic acids [20]. Among the well known supports, superparamagnetic iron oxide nanoparticles (Fe_3_O_4_; SPIONs) are of great interest, due to the easiness of obtaining them, their size and shape control, but mostly because of their magnetic properties, affording easy separation, targeting within samples and organisms and their visualization with either microscopic or resonance imagining [21,22]. Hitherto literature presents several papers devoted to the synthesis, characterization and final biomedical applications of Fe_3_O_4_ nanoparticles functionalized with PAMAM dendrimers of various structures and generations [23,24,25,26]. For instance, G3.5 PAMAM grafted on the iron oxide nanoparticles’ surface was proved to be negligibly more cytotoxic towards HeLa cells than bare particles, indicating the cytocompatibility of such systems with a simultaneous ability of penetration into bacteria cells [27]. A similar PEGylated material decorated with folic acid as a targeting residue was tested for the delivery properties of paclitaxel towards MCF-7 human breast cancer cell line, which resulted in the cancer cell viability decreasing by 34% [28]. Moreover, the efficiency of Fe_3_O_4_ particles modified with PAMAM dendrimers through various linkers has been studied in delivery of doxorubicin (versatile anticancer drug) and epigallocatechin gallate (therapeutic agent against various cancers, stroke or Parkinson’s and Alzheimer’s diseases) and has been explored towards HeLa cells [29,30,31]. The high efficiency of the drug loading of the materials and their proper cytocompatibility have been reported, which make them potential materials for therapeutics delivery with minimized side effects. Furthermore, iron oxide nanoparticles modified with citric acid and G5.0 PAMAM dendrimer have been considered to play the role of curcumin delivery platforms [32]. The material incubated with human breast adenocarcinoma (MCF-7 cell line) showed 45% cell viability at a micromolar concentration of the Fe_3_O_4_/PAMAM/curcumin system. In addition, a PAMAM dendrimer made of unusual branching units, which were 1,3-propanodiamine and glutaraldehyde, was branched around Fe_3_O_4_ nanoparticles and proved to show efficient antimicrobial activity towards two cell lines: *Escherichia coli* and *Staphylococcus Aureus* [33]. However, such hybrid materials find application not only as supports for the targeted delivery of biomolecules, but also as supporting materials for the delivery of nitrogen oxide (NO), which might be classified as an endogenous agent taking part in many physiological and pathophysiological pathways, e.g., wound healing and immune response. Yu et al. [34] have presented a method for the synthesis of *N*-diazeniumdiolate-functionalized hybrid material as a new NO-release precursor. The material was obtained in a reaction between Fe_3_O_4_/polydopamine/G3.0 PAMAM and nitrogen oxide under elevated pressure. The same material was investigated for antimicrobial activity against *E. coli* and *S. Aureus* under infrared irradiation to ensure NO-releasing conditions. The studies resulted in obtaining highly effective material with the controllable release of nitrogen oxide performed in IR switch-on/off mode.

Biological applications of the PAMAM-grafted hybrid materials based on other platforms such as single- or multi-walled carbon nanotubes [35,36,37], halloysite nanotubes [38,39,40], mesoporous silica [41,42], titania [43], gold nanorods [44], polymeric chains [45] and quantum dots made of CdSe/ZnS or CdSe/CdTe binary systems [46,47] have also been studied. Nevertheless, the pressing need for novel transporting systems has prompted us to focus on silica-based hybrid materials functionalized with PAMAM dendrimers. Our recent study was aimed at the synthesis of a series of structurally different dendritic structures with tris(2-aminoethyl)amine as a core molecule and ethylenediamine, tris(2-aminoethyl)amine, triethylenetetramine and 4,7,10-trioxa-1,13-tridecanediamine as surface amine residues. The dendrimers were introduced onto a silica surface via a linker with epoxy groups using a ‘grafting to’ approach, which allowed the retention of the fully dendritic feature of modifying agents. The obtained group of PAMAM-modified materials was investigated to establish their adsorptive properties towards folic acid, salicylic acid and nicotinic acid as the model bioactive molecules, with their further in vitro release studies under various paraphysiological conditions.

## 2. Results and Discussion

### 2.1. Synthesis of PAMAM Dendrimers

**Figure 1:**
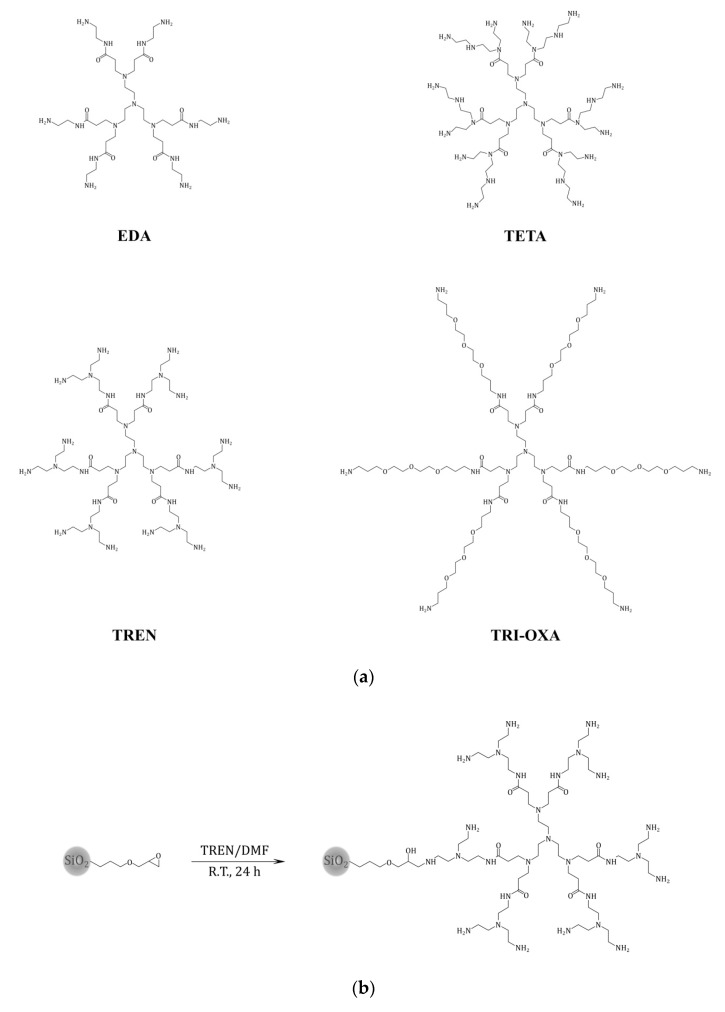
(**a**) The structures of the synthesized poly(amidoamine) (PAMAM) dendrimers containing tris(2-aminoethyl)amine as an amine core and different terminal amino-components: EDA (ethylenediamine), TETA (triethylenetetramine), TREN (tris(2-aminoethyl)amine) and TRI-OXA (4,7,10-trioxa-1,13-tridecanediamine); (**b**) the synthetic approach to obtaining hybrid materials grafted with poly(amidoamine) (PAMAM) dendrimers (on the example of tris(2-aminoethyl)amine (TREN) dendrimer grafting).

The PAMAM dendrimers consisting of tris(2-aminoethyl)amine as the amine core molecule were obtained using the two-step synthetic protocol. The first step involved the branching of the amine core molecule using the Michael addition between tris(2-aminoethyl)amine and methyl acrylate, leading to an ester intermediate, which was the precursor of desired dendritic structures. The second step was based on the amidation of the ester intermediate with proper amine, which led to obtaining each of the designed PAMAM dendrimers (Figure 1a). The choice of structurally different terminal amino-components was made on the basis of the expected influence of the terminal amine structure on the adsorptive properties of the final hybrid materials. The semi-product and the products were characterized by the electrospray ionization mass spectrometry (ESI-MS) technique. The spectrum of the ester intermediate (Figure S1) shows a highly intensive molecular peak *m/z* = 663.62 and its sodium derivative *m/z* = 685.55. On the other hand, the spectra of PAMAM dendrimers (Figure S2) show several signals corresponding to fragmentation peaks, which are strongly connected to the capability of dendrimers to fragmentation, which is a result of their chemical structure (several polyamine and carbonyl domains) and the choice of ionization mode [48]. The spectra also show several signals corresponding to sodium adducts, as well as mono-, di- or even triprotonated signals, regarding the polyamine character of the studied dendrimers.

### 2.2. Synthesis and Characterization of the Hybrid Materials

**Figure 2:**
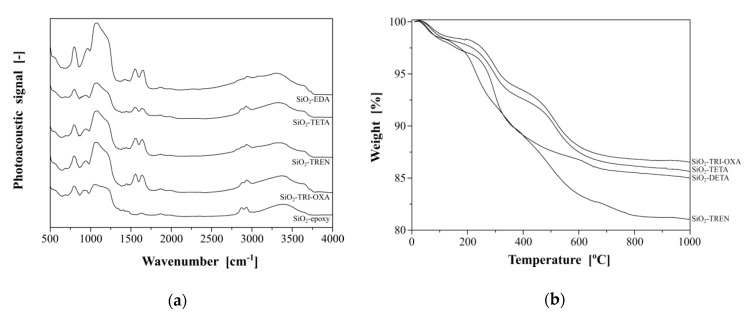
(**a**) The Fourier-transformed photoacoustic infrared (FT-IR/PAS) spectra of the PAMAM-modified hybrid materials; (**b**) the thermogravimetric analysis of the synthesized hybrid materials.

The hybrid materials functionalized with the synthesized PAMAM dendrimers were obtained in the reaction between the free terminal amine group of the dendrimer and the easy opening epoxy ring on the silica surface (Figure 1b). The choice of *N,N*-dimethylformamide (DMF) instead of the usual PAMAM dendrimers’ solvent, which is methanol, was driven by the non-nucleophilic character of DMF, decreasing the possibility of a side reaction between epoxy silica-surface groups and solvent molecules. The successful grafting of dendrimers on the silica surface was proven by Fourier-transformed photoacoustic infrared (FT-IR/PAS) spectra results (Figure 2a). All the spectra are similar, which is a consequence of the utilization of epoxy-modified silica gel as the support for PAMAM-functionalized hybrid materials. Each spectrum shows two signals assigned to the asymmetric stretching of silica matrix, which have their maxima approximately at 797 and 952 cm^−1^. Morevoer, broad bands between 1000 and 1250 cm^−1^ are related to the symmetric stretching of Si-O-Si domains. Nevertheless, the proper functionalization of silica support with particular PAMAM dendrimers is proved by the presence of signals at 1560 and 1640 cm^−1^, which correspond to the bending of N-H and the stretching of C=O, respectively, both present in amide-rich dendrimers anchored to the silica surface. These signals are not present in the spectrum of the silica precursor (SiO_2_-epoxy), which undoubtedly proves the PAMAM-functionalization of the hybrid materials. Moreover, several signals at 2889, 2936 and 2974 cm^−1^ are related to the symmetric and asymmetric stretching of methylene groups -CH_2_- present in the studied dendritic structures. These signals are noticeable on each spectrum, which might be attributed to the presence of multiple methylene groups in PAMAM dendrimers (the spectra of the hybrid materials) or to the methylene groups originating from the propylosilane epoxy-linker (SiO_2_-epoxy). In addition, the broad bands between 3050 and 3450 cm^−1^ in the hybrid materials spectra can be attributed to the overlapped stretching modes of N-H and O-H vibrations, originating in multiple terminal primary amine groups and free hydroxyl groups remaining after the functionalization of silica by the epoxy-ring opening [49]. The broad signal in SiO_2_-epoxy, however, may originate in free hydroxyl groups as a result of trace humidity in the material leading to the ring opening. The successful functionalization of silica particles with PAMAM dendrimers was also indicated by the thermogravimetric analysis (Figure 2b), which showed the samples’ mass loss up to 20%. Each spectrum shows two distinct decomposition steps. The first one is visible upon heating to around 200 °C and is strictly connected to the trace humidity removal from the samples studied. This step is manifested as approximately 2.5% mass loss of the samples and it is followed by the slow oxidation of the organic residue anchored to the inorganic platforms. This thermal decomposition takes place in the temperature range between 250 and 700 °C, leading to 10–16% mass loss of the samples.

**Figure 3:**
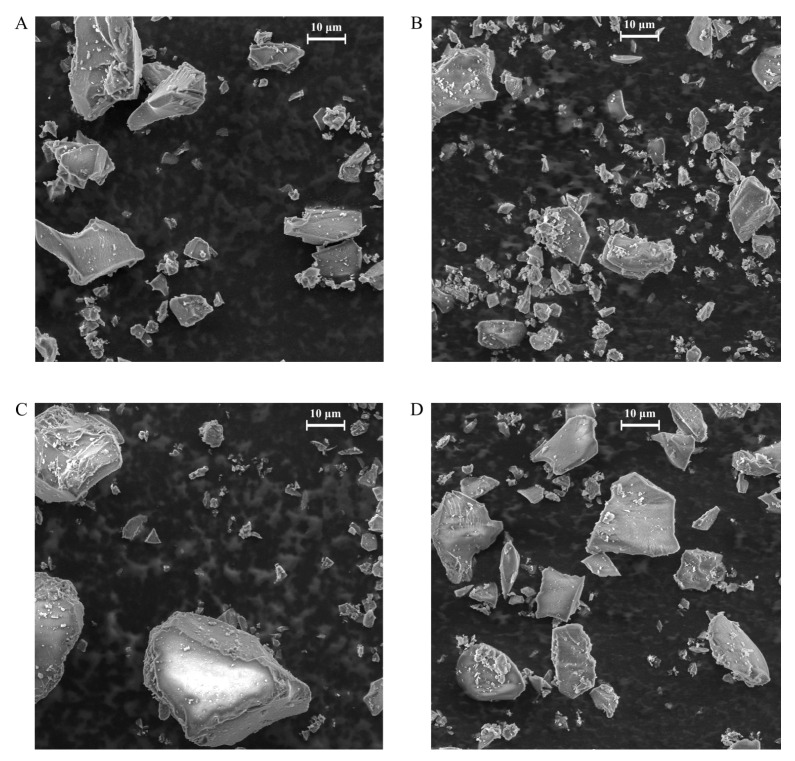
The SEM images of the obtained PAMAM-modified hybrid materials: (**A**) SiO_2_-EDA, (**B**) SiO_2_-TETA, (**C**) SiO_2_-TREN and (**D**) SiO_2_-TRI-OXA. The scale bar is 10 μm.

Moreover, the hybrid materials obtained were subjected to the CHN elemental analysis to assess the percentage contents of carbon, hydrogen and nitrogen. The most informative results of the elemental analysis were those of the nitrogen percentage contents, as carbon and hydrogen appear also in the glycidoxypropyl linker between the silica matrix and the grafted PAMAM dendrimers. The nitrogen percentage content was 2.521%, 2.122%, 3.196% and 2.010% for SiO_2_-EDA, SiO_2_-TETA, SiO_2_-TREN and SiO_2_-TRI-OXA, respectively. The loadings of dendritic structures on the silica support were calculated on the basis of these values to be 0.113, 0.054, 0.082 and 0.094 mmol g^−1^, respectively, so in good agreement with approximations following from the thermogravimetric analysis. The highest loading efficiency was calculated for the material containing surface EDA dendrimer molecules, which consist of the most compact and the least branched terminal amine used—ethylenediamine. On the other hand, the lowest loading values were calculated for the materials functionalized with TETA and TREN dendrimers with amine terminal domains whose branched character was defined before the synthesis (tris(2-aminoethyl)amine) or was a consequence of the polyamine structure that determined the differentiation in the reactivity of primary and secondary amine groups (triethylenetetramine). Furthermore, Figure 3 presents the SEM images of the synthesized hybrid materials. All the silica gel particles can be classified as microparticles with the mean size of approximately 40 μm. The functionalization of the support with more branched dendrimers TETA and TRI-OXA led to a formation of the particles with a higher mean size, as a result of the bulky dendritic structures (Figure 3C,D). The silica precursor used was described as a porous, micro-sized material, therefore the attachment of the dendrimers did not greatly enhance the size of the final hybrid materials.

### 2.3. Adsorption of Bioactive Compounds on the Hybrid Materials

**Figure 4:**
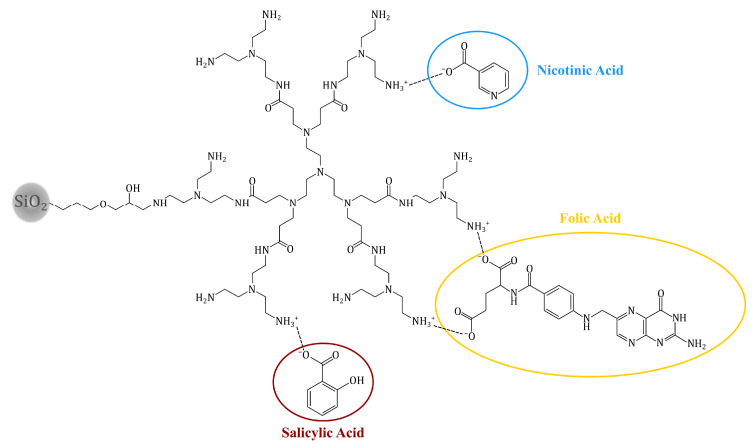
The possible electrostatic interactions (dotted line) between the PAMAM-grafted silica particles and bioactive compounds, the example of SiO_2_-TREN material.

The adsorptive properties of the obtained materials were tested for three bioactive compounds: folic, salicylic and nicotinic acids. The choice was driven by their highly broad application as drugs and vitamin supplements, while folic acid is also a preferable candidate for drug-delivery investigation, as it is chemically similar to the broadly used anticancer agent, methotrexate. Moreover, the chosen bioactive compounds are structurally different, which may bring preliminary information of the relationship between the adsorption ability and structure of the analyte. The investigation of the SiO_2_-PAMAM adsorption properties was based on the possibility of proton exchange between the highly basic PAMAM dendrimers on the silica surface and the acidic adsorbate molecules. Such an exchange, leading to the formation of a cationic form of the dendrimers (-NH_3_^+^) and an anionic form of biomolecules (-COO^–^), enables effective electrostatic interactions between the hybrid materials and the adsorbates. The ESI-MS spectra of the complexes of exemplary PAMAM dendrimers containing tris(2-aminoethyl)amine as the terminal amine and each of the bioactive compounds used (Figure S3) proved the proton exchange between the tested components. The ESI-MS spectra of TREN-salicylic acid (Figure S3a) and TREN-nicotinic acid (Figure S3b) in positive mode showed signals originating mostly from the monoprotonated forms (z 1+) of the dendrimers’ fragmentation products (the signal at *m*/*z* 1201.98, 1055.84, 983.20, 909.70), which strictly indicated the single proton exchange between the dendrimer and the drug. The corresponding spectra in negative mode presented the most intensive signals related to the deprotonated forms of the salicylic and nicotinic acids at *m*/*z* 137.36 and *m*/*z* 122.45, respectively. Moreover, several mono-negative signals (z 1−), corresponding to the adducts of the fragmented -COOH domain of biomolecules and the fragmented dendritic structures appear, e.g., at *m*/*z* 570.47, 643.55, 907.61, 951.59, 1097.77 or 1243.93, which are similar in both the TREN-salicylic acid and TREN-nicotinic acid complex spectra. In the case of the TREN-folic acid (Figure S3c), an exchange of the two protons derived from dicarboxylic drug takes place, determining the appearance of mostly di-, but also tri- or even tetra-positive signals in the positive ESI-MS spectrum corresponding to the dendrimers’ fragmentation ions or their sodium adducts (*m*/*z* (z 2+) 612.65, 601.68, 539.60, 528.65, 466.53, 455.55; *m*/*z* (z 3+) 401.61, 352.87; *m*/*z* (z 4+) 301.64). The least intensive signals at *m*/*z* 909.64 and 1055.81 related to the monoprotonated (z 1+) fragmentation ions of the dendrimer might be a consequence of the single proton exchange between the folic acid and TREN dendrimer. In the negative spectrum, the most intensive signals are related to the di-negative ion (z 2−) of folic acid *m*/*z* 219.69, however the spectrum also exhibits several mono-, di- and tri-negative signals at *m*/*z* 1243.98, 1097.74, 951.55, 644.08, 570.48, 544.82, 462.80 or 380.89, corresponding to the adducts of the fragmented dendrimer and the fragmented carboxylic group of biomolecules. There is also a mono-negative signal at *m*/*z* 440.23, which is connected with the exchange of a single proton, which when interpreted together with the previous results proves the formation of the dendrimer-drug complex, indicating the PAMAM-grafted materials’ capability of binding the acidic drugs, which is shown in Figure 4. Therefore, the ESI-MS analysis showed that the chosen drugs can be bound to surface dendritic structures only via electrostatic interactions with terminal amine groups. Although, the amine groups are also utilized in the dendrimers’ immobilization process, the dendrimers may easily interact with analytes through the other multiple free -NH_2_ domains.

**Figure 5:**
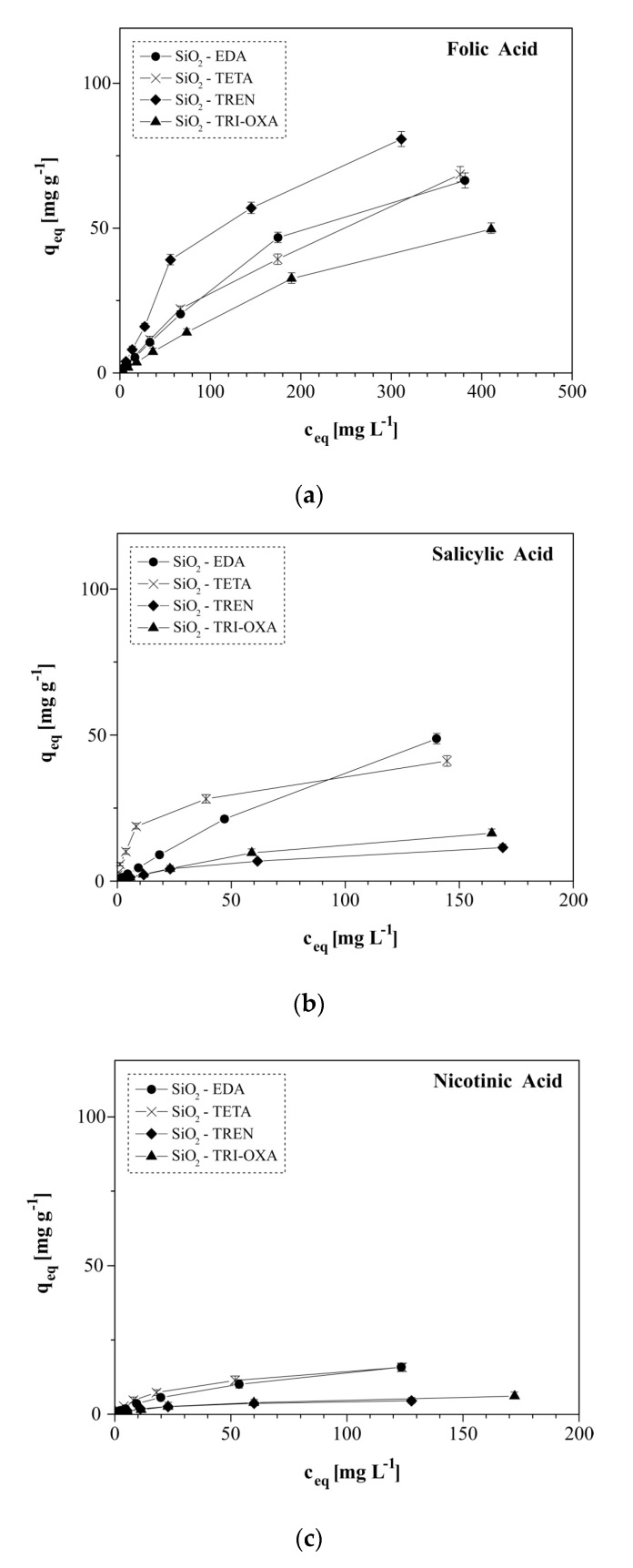
The isotherms of the acidic drugs’ adsorption on the obtained hybrid materials: (**a**) folic acid; (**b**) salicylic acid; and (**c**) nicotinic acid. For some points, the SDs are smaller than the plotted symbols.

The adsorptive properties of the obtained PAMAM-grafted materials were tested in isothermal studies (Figure 5), which delivered information about the behavior of adsorbent-adsorbate interactions and their efficiency at equilibrium state. Therefore, the experimental data were fitted to several adsorption isotherm models for the assessment of particular physicochemical quantities. The most broadly used models are the Langmuir and Freundlich ones, which take into account the distribution of the binding places on the adsorbents’ surface and the interactions between the adsorbate molecules [50]. The Langmuir model (Figure S4) assumes the formation of the adsorbate monolayer on the homogenous adsorbent surface, neglecting the adsorbate molecules’ interaction, and can be expressed by Equation (1), where c_eq_ is the concentration of the drug remaining in the solution at equilibrium (mg L^−1^), q_eq_ and q_max_ are the equilibrium and the maximal amount of the drug adsorbed, respectively (mg g^−1^), while K_L_ is the Langmuir constant (L mg^−1^):

(1) $$\frac{c_{\mathrm{eq}}}{q_{\mathrm{eq}}}=\frac{c_{\mathrm{eq}}}{q_{\max}}+\frac{1}{q_{\max}K_{L}}$$

In contrast, the Freundlich isotherm model (Figure S5) indicates the possibility of adsorbate multilayer formation, which is a consequence of the interactions of adsorbate molecules. Moreover, it assumes the heterogeneity of the adsorbent surface, influencing the adsorptive properties of materials, and is expressed as Equation (2):

(2) $$\log q_{\mathrm{eq}}=\frac{1}{n}\log c_{\mathrm{eq}}+\log K_{F}$$

where 1/n is the coefficient related to the heterogeneity of the adsorptive material (−) and K_F_ is the Freundlich constant (mg g^−1^ (L mg^−1^)^1/n^). On the basis of both aforementioned linear plots of the isothermal models, all the parameters and their standard deviations can be easily calculated from slopes and intercepts; the relevant values are collected in Table 1.

All the materials, including the SiO_2_-epoxy material, which is a bare precursor of the synthesized hybrid materials, exhibit an adsorption capacity towards all the biocompounds studied, which is in agreement with the Freundlich isothermal model, as confirmed by the R^2^ correlation coefficients higher than 0.985 for all the experiments. It is also indicated in Table 1, where the R^2^ values for the Freundlich model are higher or equal to the R^2^ values calculated for the Langmuir model, that the χ^2^ values calculated for the Freundlich model are mostly lower than those calculated for the Langmuir fitting. Therefore, the adsorption of the biocompounds studied on the materials obtained is possible thanks to the materials’ surface heterogeneity, proved by the Freundlich 1/n factors values ranging between 0 and 1 and also the intermolecular interactions between the adsorbate molecules, which are easily proved by the structural features of the bioactive compounds used. Namely, folic acid contains three aromatic rings, which may easily interact with each other by the π-π stacking effect, as well as electrostatic interactions between two carboxylic groups and several amine groups (terminal, internal or even the one contained in pteridyl domain), while nicotinic and salicylic acids may be engaged in intermolecular interactions thanks to the presence of aromatic rings in both structures. Therefore, folic acid is the most intensively adsorbed, independently of the material used. Moreover, the amount of hydrogen-bonding acceptors and donors in the biocompounds’ structure, and also their pK_a_ values, influence the intensity of the interaction with basic PAMAM dendrimers. The ratio of the hydrogen-bonding acceptors to donors is 10/6 for folic acid, 3/2 for salicylic acid and 3/1 for nicotinic acid, which firmly indicates the formation of the strongest interactions between the basic PAMAM dendrimers and folic acid, while the weakest is with nicotinic acid. In addition, the acid-base interaction of the surface dendrimers with biocompounds is dependent on their pK_a_ values, namely the lower the pK_a_ value, the stronger the interaction. The pK_a_ values are 3.49 for folic acid, 2.97 for salicylic acid and 4.75 for nicotinic acid. On the basis of the mentioned chemical features of the studied bioactive acids, the biocompounds can be ordered according to their binding efficiency as: folic acid > salicylic acid > nicotinic acid. Nevertheless, the reliable fitting of the experimental data to the Langmuir isothermal model led to the determination of maximal adsorption capacities (q_m_) for all the hybrid and bare materials. As expected, SiO_2_-epoxy material (bare silica support) showed the lowest adsorption capacity towards the bioactive acids among all the adsorbents, reaching approximately 12.5 mg g^−1^ for folic acid, and 1.5 and 1.3 mg g^−1^ for salicylic and nicotinic acid, respectively. The binding of therapeutic substances in the material that does not contain dendrimer as the domain biding through electrostatic interactions of the physical entrapment is thus based on the physical diffusion of drugs into the material pores. SiO_2_-EDA which contains the dendrimer having the shortest and the least branched terminal aminocomponent used, ethylenediamine, turned out to be the most effective adsorbent of all the acidic bioactive compounds studied. The use of ethylenediamine ensured the highest dendrimer-grafting efficiency at the level of 0.113 mmol g^−1^, which contributed to the significantly increased adsorption capacity of the material containing it, towards the bioactive acidic compounds, reaching the maximal adsorption capacities of 274.72, 157.48 and 20.11 mg g^−1^ for folic, salicylic and nicotinic acids, respectively. A high-adsorption efficiency was also noted for the SiO_2_-TREN material, containing a surface dendritic structure built of symmetric tris(2-aminoethyl)amine. The doubled number of the ultimate interacting amine groups and the increased number of cavities in the interior of the dendrimer should have significantly enhanced the q_m_ values, however, the steric hindrance of the TREN dendrimer contributed to its lower loading onto the silica surface (0.082 mmol g^−1^), which jointly led to high, but not the highest, adsorption effectiveness. The least adsorptive materials were the ones decorated with the TETA and TRI-OXA dendrimers, which might be a consequence of steric hindrance and the low functionalization percentage of the dendritic structures. In addition, the long-chained dendrimer containing additional oxygen atoms (TRI-OXA dendrimer) may not be capable of the effective binding of compounds because of the presence of a long methylene-rich chain which does not improve the interaction with acidic adsorbates. Thus, the adsorptive efficiency of the materials may be ordered as: SiO_2_-EDA > SiO_2_-TREN > SiO_2_-TETA ~ SiO_2_-TRI-OXA. Moreover, for all the adsorption experiments, the dimensionless separation Langmuir factors R_L_ were calculated using Equation (3), where c_max_ is the maximal concentration of the drug solutions used for the isothermal experiments (mg L^−1^):

(3) $$R_{L}=\frac{1}{1+K_{L}c_{\max}}$$

For all the experiments, the R_L_ factors ranged between 0 and 1, which indicates the favorability of the adsorption processes [51]. Furthermore, the experimental data were fitted to the Temkin and Dubinin-Radushkevich isothermal models, which are strictly connected with specific values of the energy of adsorption, therefore making useful tools for the investigation of physical or chemical characters of adsorptive processes studied [50]. The Temkin isotherm linear plot (Figure S6) is given by Equation (4), where K_T_ is the Temkin isotherm constant, while B is the Temkin constant strictly related to the heat of adsorption:

(4) $$q_{\mathrm{eq}}=B\ln c_{\mathrm{eq}}+B\ln K_{T}$$

Specific values, which determine whether chemi- or physisorption takes place, may be calculated using the linear plot of the Dubinin-Radushkevich isotherms model (Figure S7) presented below (Equation (5)), which is described by ln q_eq_ vs. ε^2^, where ε is the Polanyi potential (J mol^−1^), calculable as ε = RT ∙ ln (1+1/c_eq_); R is the ideal gas constant (8.314 J mol^−1^K^−1^), and T is the temperature of the process:

(5) $$\ln q_{\mathrm{eq}}=-\beta \ln\varepsilon^{2}+\ln q_{m}$$

According to the above plot, the slope of the linear plot, which equals to the activity coefficient β (mol^2^ J^−2^), is necessary for the determination of the mean energy of adsorption *E* (J mol^−1^) using Equation (6):

(6) $$E=\sqrt{\frac{1}{-2\beta }}$$

The calculated values of the Temkin constants *B* (Table S1) show that the adsorption energy is significantly smaller for the precursor material (SiO_2_-epoxy) than for the hybrid materials functionalized with PAMAM dendrimers. These parameters also indicate the physical character of sorption processes as *B* values are lower than 20 J mol^−1^ for each experiment. Physisorption is also proved by the calculated mean energy of the adsorption value *E* (Table S1), which varies between 0.02 and 2.45 kJ mol^−1^ for all the conducted experiments, remaining lower than 8 kJ mol^−1^, which is a limit for physical sorption [52].

### 2.4. Thermodynamic Studies of the Drug Adsorption Processes on the Hybrid Materials

The processes of complex formation between the chosen bioactive acids and the hybrid materials were also described with thermodynamic parameters. Therefore, thermochemical parameters such as the adsorption enthalpy ∆*H*° and the adsorption entropy ∆*S*° may be determined using an appropriate principle. The van’t Hoff equation, given below as Equation (7), shows a relation between these parameters and the temperature:

(7) $$\ln K_{d}=-\frac{\Delta H{}^{\circ}}{\mathrm{RT}}+\frac{\Delta S{}^{\circ}}{R}$$

where: K_d_ is the equilibrium constant defined as the ratio of the amount of the drug adsorbed at equilibrium to the concentration of the drug remaining in the solution (K_d_ = q_eq_/c_eq_) (L g^−1^), T is the temperature (K) and R is the ideal gas constant (8.314 J mol^−1^ K^−1^). From the slope and intercept of the linear plot of lnK_d_ vs. 1/T (Figure S8), all the parameters are obtainable. In addition, Gibbs free energy values of the biomolecules’ adsorption process at different temperatures were calculated using Equation (8):

(8) $$\Delta G{}^{\circ}=-\mathrm{RT}\cdot \ln K_{d}$$

The calculated thermodynamic parameters are collected in Table 2. High-correlation coefficients R^2^ and extremely low χ^2^ values highlight the reliability of the obtained energy values. Very low and positive adsorption enthalpies, which for most of the adsorption processes are lower than 15 kJ mol^−1^, clearly indicate the endothermic physisorption, which is stabilized only by non-covalent binding, such as electrostatic interactions and hydrogen bonds [53]. In addition, the adsorption processes are driven by the entropy, whose positive values indicate the increased randomness in the adsorbent-solution interface with the temperature rise. Moreover, the endothermic character of bioactive acids’ adsorption is proved by the decrease in negative ∆*G*° values with increasing temperature, reaching the lowest values at 328 K indicating the highest intensity of adsorption (∆*G*°_328K_ < ∆*G*°_313K_ < ∆*G*°_301K_). Their negative values also indicate the spontaneity of the processes.

### 2.5. pH-Dependent Drug Release from the Drug-Loaded Hybrid Materials

The release of drugs anchored to the surface of the hybrid materials studied was performed in three different media: phosphate buffered saline (PBS) (pH 7.4), acetic acid/sodium acetate buffer (pH 5.4) and hydrochloric acid/potassium chloride buffer (pH 2.0). The choice of such environments was driven by the different pH values of blood, plasma and saliva (pH 7.4); skin (pH 5.5); and gastric juice (pH 1.5–3.0). Moreover, the main interactions taking part in the material-drug complexes are electrostatic ones, which are labile towards pH changes and ionic strength. In vitro release experiments were held at 37 °C, imitating paraphysiological conditions.

**Figure 6:**
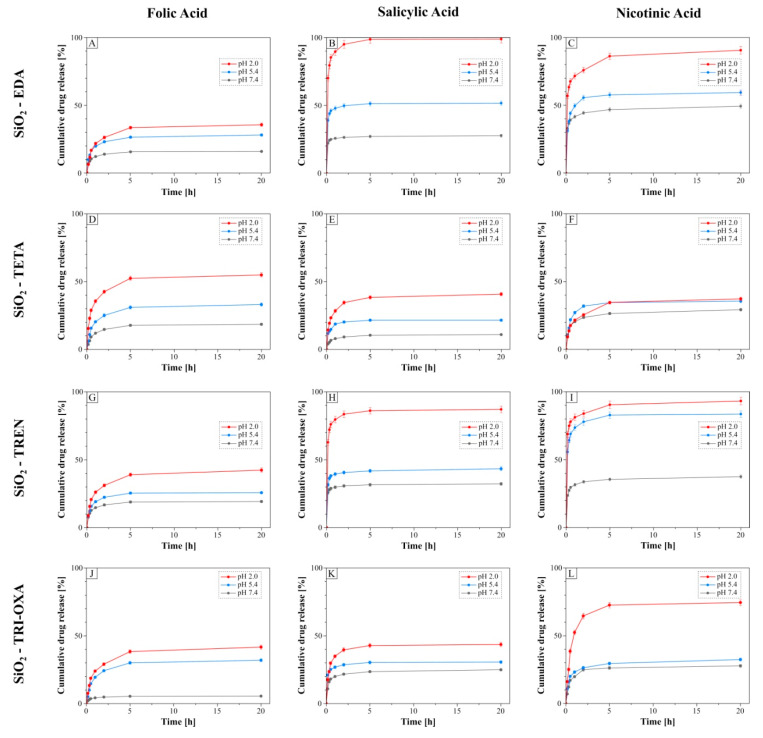
Release profiles of the biocompounds (folic acid: **A, D, G, J**; salicylic acid: **B, E, H, K**; nicotinic acid: **C, F, I, L**) from the drug-loaded hybrid materials: SiO_2_-EDA (**A**–**C**); SiO_2_-TETA (**D**–**F**), SiO_2_-TREN (**G**–**I**), SiO_2_-TRI-OXA (**J**–**L**) in three different environments. For some points, the SDs are smaller than the plotted symbols.

Figure 6 presents the release profiles of folic acid (A, D, G, J), salicylic acid (B, E, H, K) and nicotinic acid (C, F, I, L) from the PAMAM-grafted hybrid materials as a function of the cumulative percentage of drug released Q_t_ in 20 h of incubation. From among all the experiments, the ones performed in the most acidic environment, which was HCl/KCl buffer at pH 2.0, gave the highest release levels ranging between 37.22% and 99.83%. The main reason for such a phenomenon is connected with introducing the excess of H^+^, which readily binds with negatively charged acidic residue, leading to repulsive interactions with the protonated amine groups of PAMAM dendrimers grafted on the silica supports. On the other hand, the lowest percentage of drug released were established for the desorption processes held in the PBS buffer. A composition of such a buffer was based on the sodium and potassium cations, and the hydrogen phosphate and chloride anions, whose proportion matches the ionic strength and osmolarity of human plasma. These ions are easily exchanging between protonated amine groups of surface PAMAM dendrimers and deprotonated biocompounds’ molecules, which does not afford the highest effectiveness of electrostatic interaction disruption, hindering the drugs’ desorption. Nevertheless, the percentage amount of drugs released from the drug-loaded hybrid materials at pH 7.4 ranges between 5.45% and 49.28% for all the experiments performed. The analysis of the relation between the chemical structure of the drugs and their release profiles has shown that the lowest efficiency of desorption was obtained for folic acid. The strongest binding affinity of folic acid to the support and the acid’s steric hindrance may both hinder its elution to an aqueous medium, irrespectively of the solution pH, thus the desorbed folic acid molecules are usually contained in external layers of the adsorbate multilayer. Therefore, the maximal folic acid releases at pH 2.0 are 35.42%, 54.90%, 42.40% and 41.59% from SiO_2_-EDA (Figure 6A), SiO_2_-TREN (Figure 6D), SiO_2_-TETA (Figure 6G) and SiO_2_-TRI-OXA (Figure 6J), respectively. Salicylic and nicotinic acid, however, have significantly smaller molecules than folic acid and exhibit a weaker binding affinity to the PAMAM-grafted hybrid materials, which allows their greater release to aqueous solutions. The highest percentage of released salicylic and nicotinic acid was calculated for SiO_2_-EDA (Figure 6B,C) and SiO_2_-TREN (Figure 6H,I), which is strictly connected with the high dendrimer grafting values of these materials and the presence of tight internal cavities within the dendrimers’ interior, jointly improving the sorption parameters. The experiments conducted in the acidic environment in pH 2.0 gave very high release percentage values ranging between 86.88% and 99.83%. On the other hand, SiO_2_-TETA, containing a TETA dendrimer grafted on the surface (branched and with larger cavities) may tightly bind salicylic and nicotinic acid by both electrostatic/hydrogen bonding and physical entrapment, hindering their elution, leading to maximal release percentage values at 40.75% for salicylic acid (Figure 6E) and 37.22% for nicotinic acid (Figure 6F). A similar observation was made when studying the release of salicylic acid and nicotinic acid from SiO_2_-TRI-OXA. The profiles of their release are shown in Figure 6K,L. The exception was the release of nicotinic acid from the same material at pH 2.0, which was much stronger than at pH 5.4 and 7.4, which can be explained by a considerable excess of protons in the HCl/KCl buffer. This excess of protons permits the effective desorption of nicotinic acid from the complex.

In order to propose a mechanism which triggers the drug release from the PAMAM-grafted hybrid materials, the experimental in vitro release at pH 2.0 (the highest release efficiency) was fitted to several kinetic models [54,55]: the zero-order model, the first-order model, the Higuchi model, the Korsmeyer-Peppas model and the Hixson-Crowell model, whose linear representations are given below in the same order:

(9) $$F_{t}=F_{0}+k_{0}t$$

(10) $$\log\left(100-Q_{t}\right)=-k_{1}t$$

(11) $$Q_{t}=k_{H}\sqrt{t}$$

(12) $$\log Q_{t}=n\log t+\log k_{K-P}$$

(13) $$\sqrt[{3}]{F_{0}}-\sqrt[{3}]{F_{t}}=k_{H-C}t$$

where: F_t_ is the cumulative amount of the drug released at time t (mg), F_0_ is the initial amount of the drug loaded in the hybrid material (mg), k_0_ is the zero-order release constant (mg h^−1^), Q_t_ is the cumulative percentage of the drug release at time t (%), k_1_ is the first-order release constant (% h^−1^), k_H_ is the Higuchi release constant (% h^−1/2^), n is the Korsmeyer-Peppas exponent of release (-), k_K-P_ is the Korsmeyer-Peppas release constant (% h^−1^), and k_H-C_ is the Hixson-Crowell release constant (mg^1/3^ h^−1^).

Table 3 presents a comparison of the experimental data fit to the first-order, the Higuchi and the Korsemeyer-Peppas release models, which are most extensively used for the description of drug release from various delivery platforms. The highest correlation coefficients R^2^ of the bioactive acids release from the PAMAM-functionalized silica materials were calculated for the Korsmeyer-Peppas release model and were equal to or higher than 0.8. Moreover, the experimental data fitted to this kinetic model were described with statistical χ^2^ coefficients, the values of which were determined to be the lowest among the values calculated for fitting to the Higuchi and the first-order kinetic models. In addition, in this model, the n value is strictly dependent on the mechanism of drug release and if n is lower than 0.45, the release follows a Fickian or quasi-Fickian diffusion model [56]. Such a conclusion can be drawn for the experiments of bioactive acids released from the materials studied, which gave n values lower than 0.34, indicating the quasi-Fickian diffusion as a mechanism of folic, salicylic and nicotinic acid release from the dendrimer-grafted materials. In addition, the experimental data were fitted to the zero-order and the Hixson-Crowell models (Table S2), however the obtained R^2^ values reached maximally 0.62, which clearly shows that the release profiles do not follow these models.

## 3. Materials and Methods

### 3.1. Materials

The support 3-(glycidoxy)propyl-functionalized silica-gel (SiO_2_-epoxy) was obtained from SiliCycle Inc. (Quebec, QC, Canada). According to the specification card, the particle sizes ranged between 40 and 63 μm, the molecular loading (grafting level) was 1.21 mmol g^−1^, the specific surface area equaled 494 m^2^ g^−1^, the pore diameter was 60 Å and pore volume was 0.74 mL g^−1^. All the reagents were commercially available products used with no further purification. Ethylenediamine, triethylenetetramine, tris(2-aminoethyl)amine, 4,7,10-trioxa-1,13-tridecanediamine, methyl acrylate, folic acid, salicylic acid, nicotinic acid were purchased from Sigma-Aldrich (St. Louis, MO, USA). All the solvents and salts used for the buffers preparation were of the purity grade p.a. Methanol and hydrochloric acid (HCl) were purchased from STANLAB (Lublin, Poland). *N,N*-dimethylformamide (DMF), dichloromethane (DCM), acetic acid (AcOH), sodium acetate (AcONa) and potassium chloride (KCl) were obtained from Eurochem (Tarnow, Poland). Di- and monosodium phosphates (Na_2_HPO_4_ and NaH_2_PO_4_) were purchased from POCH (Gliwice, Poland).

### 3.2. Instruments

The ESI-MS spectra of the dendrimers and the dendrimer-biocompound complexes were recorded on amaZon SL ion trap Bruker mass spectrometer (Bremen, Germany), using electrospray ion source (ESI) in infusion mode. Sample solutions were introduced to the spectrometer using a syringe pump, at a flow rate of 10 μL min^−1^ into the ionization source. Analyses were performed in the so-called “enhanced resolution mode” with mass ranges between 100 and 2200 *m*/*z*. Capillary voltage was determined at −4.5 kV and −500 V for the endplate offset. The source temperature was set at 80 °C and the desolvation temperature at 250 °C. Helium and nitrogen were used as the cone and the desolvating gases, respectively. The flow rate of helium was set at 50 L h^−1^ and 800 L h^−1^ for nitrogen. The Fourier-transformed photoacoustic infrared (FT-IR/PAS) spectra of the synthesized hybrid materials were obtained by means of Bio-Rad Excalibur FTIR 3000 MX spectrometer (Hercules, CA, USA), using the MTEC Model 300 photoacoustic cell and carbon black standard as a reference sample. Before the data collection within the wavenumber range between 4000 and 400 cm^−1^, the cell was purged with dry helium. The thermogravimetric measurements were performed using Setaram Setsys 1200 analyser (Caluire, France). The hybrid materials were investigated for thermal stability in air stream within the temperature range of 20–1000 °C (a heating rate: 5 °C min^−1^). The elemental analyses of the materials were carried out in Elementar Vario EL III analyzer (Langenselbold, Germany) in CHN mode. The SEM images of the hybrid materials were performed using the Quanta FEG 250 scanning electron microscope. The images were recorded in high vacuum conditions (1.21–5.33∙10^−3^ Pa) and high voltage, set at 5 kV. The working distance during the image production ranged between 9.7 and 10.1 mm. Concentration of the drugs in the samples of adsorption and in vitro release studies was determined by spectrophotometric method, using the Agilent 8453 UV-Vis spectrophotometer (Santa Clara, CA, USA). All the measurements were performed at least in triplicate, in order to overcome disturbances.

### 3.3. Synthesis of PAMAM Dendrimers

To a solution of methyl acrylate (12.23 mL, 0.135 mol) in 50 mL of anhydrous methanol, cooled to temperature 0 °C, a solution of tris(2-aminoethyl)amine (4.49 mL, 0.03 mol) of in 30 mL of anhydrous methanol was added dropwise within 2 h, under nitrogen atmosphere, not to allow temperature rise above 5 °C. Then, the reaction mixture was allowed to warm to room temperature and stirred for 5 days. Afterwards, the solvent and the excess of methyl acrylate were evaporated, and the product was dried under vacuum at 40 °C. The orange liquid ester intermediate was obtained with 98% yield. ESI-MS: *m*/*z* (z 1+) 663.62, 685.55.

The synthesis of final PAMAM dendrimers was performed according to the following procedure: to the solutions of particular amines cooled to the temperature of 0 °C: ethylenediamine (3.00 mL, 45 mmol), triethylenetetramine (6.71 mL, 45 mmol), tris(2-aminoethyl)amine (6.73 mL, 45 mmol) or 4,7,10-trioxa-1,13-tridecanediamine (9.95 mL, 45 mmol) in 50 mL of anhydrous methanol, the solution of ester intermediate (3.31 g, 5 mmol) in 20 mL of anhydrous methanol was added dropwise within 2 h not to let the temperature rise above 5 °C. Afterwards, the mixtures were warmed to room temperature and stirred for 5 days in the atmosphere of inert gas (N_2_). The crude products were obtained by extraction with cold diethyl ether (3 × 30 mL). The products were then evaporated and dried under vacuum at 40 °C, obtaining pure EDA, TETA, TREN and TRI-OXA dendrimers, respectively.

**EDA:** yield = 95%; ESI-MS: *m*/*z* (z 1+) 831.78, 771.71, 717.73, 657.68, 603.69

**TETA:** yield = 71%; ESI-MS: *m*/*z* (z 1+) 1255.94, 1227.94, 1201.99, 1174.02, 1147.99, 1081.84, 1055.83, 1027.86, 1001.84, 973.86, 909.69, 855.73; *m*/*z* (z 2+) 628.63, 614.67, 601.68, 587.68, 574.67, 528.58, 501.57

**TREN:** yield = 83%; ESI-MS: *m*/*z* (z 1+) 1348.11, 1223.96, 1201.99, 1077.83, 1055.84, 931.68, 909.72; *m*/*z* (z 2+) 612.69, 601.71, 539.60, 528.62

**TRI-OXA:** yield = 91%; ESI-MS: *m*/*z* (z 1+) 1792.11, 1682.47, 1572.96, 1518.06, 1297.95; *m*/*z* (z 2+) 897.17; *m*/*z* (z 3+) 598.36

### 3.4. Synthesis of Hybrid Materials

Briefly, 2 mmol of each dendrimer (1.6 g EDA; 2.7 g TREN; 2.7 g TETA or 3.6 g TRI-OXA) was dissolved in 50 mL of anhydrous DMF, and then 5 g of silica modified with glycidoxypropyl linker (1.21 mmol g^−1^; 6.05 mmol of epoxy groups) were added in a few portions under a nitrogen atmosphere. The stirring was continued for 24 h at room temperature. Afterwards, the solids were filtered off and washed with DMF (2 × 10 mL) and DCM (1 × 15 mL). The pale yellow solids were dried in a vacuum at 40 °C, obtaining SiO_2_-EDA, SiO_2_-TETA, SiO_2_-TREN and SiO_2_-TRI-OXA materials, respectively.

### 3.5. Biomolecules Adsorption Isotherms

Isothermal studies were performed individually for each hybrid material towards each of the three bioactive compounds, i.e., folic, salicylic and nicotinic acid. The general procedure was as follows: 10 mg samples of the hybrid material were poured into a series of 10 mL of bioactive compound aqueous solutions with various concentrations: 0.01, 0.05, 0.1, 0.2, 0.5 and 1 mM. Salicylic and nicotinic acids were dissolved in distilled water, while folic acid in phosphate buffer at pH 8.0. The materials were stirred in the drug solutions for 24 h at room temperature. Afterwards, the solids were filtered off and the amount of the drug adsorbed was calculated using Equation (14):

(14) $$q_{\mathrm{eq}}=\left(c_{0}-c_{\mathrm{eq}}\right)\cdot \frac{V}{m}\cdot M$$

where: c_0_ is the starting concentration of the biomolecule (mM), c_eq_ is the equilibrium concentration of the biomolecule that remained in the solution (mM), V is the volume of the biomolecule solution (mL), M is the molar mass of the biomolecule (g mol^−1^), and m is the mass of the hybrid material sample (mg). The concentrations of the drug remaining in the solution were calculated using UV-Vis spectrophotometric assays with absorption maxima at 365, 297 and 263 nm for folic, salicylic and nicotinic acid, respectively.

### 3.6. Thermodynamic Studies of Biomolecules Adsorption on the Hybrid Materials

Thermodynamic studies were also conducted for each of the hybrid samples towards each drug, individually. The general procedure was based on the stirring of 10 mg samples of the hybrid material in 10 mL of 1 mM solution of particular biocompound (salicylic or nicotinic acid dissolved in distilled water, while folic acid was dissolved in phosphate buffer at pH 8.0) at 298, 313 and 328 K for 24 h. Then, the materials were filtered off and the amount of the acid adsorbed was calculated as described in Section 3.5.

### 3.7. In-Vitro Release of Bioactive Compounds

The hybrid materials loaded with either folic, salicylic or nicotinic acid were tested for the release of the drug in three different environments: pH 2.4 (hydrochloric acid/potassium chloride buffer), pH 5.4 (acetic acid/sodium acetate buffer) and pH 7.4 (phosphate buffered saline: PBS). The general procedure involved the incubation of 15 mg of the drug-loaded materials in 5 mL of the desorbing aqueous solutions at a temperature of 37 °C. The buffers aliquots were collected at the pre-set time intervals, with the subsequent addition of 5 mL of fresh buffers. The cumulative percentages of the drug released Q_t_ at time t were calculated using Equation (15), where c_t_ is the concentration of the drug released at time t (mM), V is the volume of the desorbing solution (mL), M is the molar mass of the drug (g mol^−1^), and m is the amount of the drug in the material-drug complex used (mg):

(15) $$Q_{t}=\frac{\sum_{i=0}^{t}\left(c_{t}\cdot V\cdot M\right)}{m}\cdot 100\%$$

The drug concentrations were calculated using UV-Vis spectrophotometric measurements of folic, salicylic and nicotinic acids at a wavelength of 365, 297 and 263 nm, respectively.

## 4. Conclusions

The study reported was aimed at determination of the adsorption properties of silica-PAMAM dendrimer hybrid materials towards chosen model biocompounds. The materials obtained by the anchoring of ready-made poly(amidoamine) dendrimer on the silica surface via epoxy linker (‘grafting to’ approach) were successfully characterized using several analytical techniques, indicating the dendrimers’ loading values variation between 0.054 and 0.113 mmol g^−1^. Although the dendrimers’ loading values were relatively low, the final hybrid materials exhibited binding efficiency towards chosen biocompounds, which is related to the branched and amine-rich character of the grafting agents. The highest adsorption capacity of the biocompounds studied was found for SiO_2_-EDA (274.72, 157.48 and 20.11 mg g^−1^ for folic, salicylic and nicotinic acid, respectively) and SiO_2_-TREN (236.97, 42.92 and 17.74 mg g^−1^ for folic, salicylic and nicotinic acid, respectively) materials. The adsorption performance was dependent on the structural features of the studied bioactive acids. Moreover, each drug-loaded material was investigated for the drugs’ in vitro release in three different aqueous media. The release percentage was found to be pH-dependent, which can be presented in a series pH 2.0 > pH 5.4 > pH 7.4. In addition, the amount of the drug released was affected by its chemical structure either hindering or improving its desorption from the material matrices, reaching maximal release between 35.42% and 54.90% for folic acid release, 40.75% and 99.83% for salicylic acid release, and 37.22% and 93.24% for nicotinic acid release.

The synthesized hybrid materials were found to be characterized with effective adsorption and in vitro release of the model drugs, which was possible thanks to the versatility of PAMAM dendrimer applications. The universality of SiO_2_-PAMAM complexes makes them attractive candidates for their future use as the systems for the adsorption/desorption of selected anticancer drugs as well as pre-concentration tools for bioactive water pollutants. In addition, the enhancement of the adsorptive properties as well as the biomedical applicability of the hybrid materials obtained are planned to be studied using different supports, such as iron oxide (Fe_3_O_4_) nanoparticles or biocompatible polymeric chains.

## Figures and Tables

**Table 1:** Isothermal parameters of the drugs’ adsorption on the PAMAM-modified materials.

Biomolecule	Adsorbent	Langmuir Isotherm				Freundlich Isotherm		
		q_m_ (mg g^−1^)	R_L_ (-)	R^2^	χ^2^	1/n (−)	R^2^	χ^2^
**Folic Acid**	SiO_2_-epoxy	12.46 ± 0.96	0.9966	0.9654	1.000	0.80 ± 0.03	0.9907	0.054
	SiO_2_-EDA	274.72 ± 6.87	0.9987	0.9963	0.003	0.93 ± 0.02	0.9979	0.017
	SiO_2_-TETA	230.95 ± 18.18	0.9882	0.9977	0.025	0.96 ± 0.01	0.9994	0.029
	SiO_2_-TREN	236.97 ± 11.79	0.9984	0.9853	0.008	0.91 ± 0.02	0.9978	0.012
	SiO_2_-TRI-OXA	202.43 ± 4.34	0.9972	0.9972	0.727	0.93 ± 0.02	0.9982	0.010
**Salicylic Acid**	SiO_2_-epoxy	1.50 ± 0.09	0.9549	0.9827	8.739	0.50 ± 0.02	0.9887	0.072
	SiO_2_-EDA	157.48 ± 9.67	0.9956	0.9777	0.006	0.93 ± 0.02	0.9973	0.013
	SiO_2_-TETA	15.44 ± 1.17	0.9803	0.9667	0.056	0.70 ± 0.03	0.9858	0.046
	SiO_2_-TREN	42.92 ± 2.10	0.8741	0.9848	0.222	0.46 ± 0.03	0.9855	0.037
	SiO_2_-TRI-OXA	29.53 ± 1.69	0.9902	0.9807	0.104	0.84 ± 0.03	0.9914	0.028
**Nicotinic Acid**	SiO_2_-epoxy	1.33 ± 0.10	0.9610	0.9641	8.190	0.54 ± 0.02	0.9871	0.030
	SiO_2_-EDA	20.11 ± 2.25	0.8758	0.9408	0.673	0.64 ± 0.03	0.9907	0.073
	SiO_2_-TETA	4.86 ± 0.25	0.8499	0.9870	1.245	0.54 ± 0.03	0.9866	0.052
	SiO_2_-TREN	17.74 ± 1.08	0.7724	0.9816	0.353	0.42 ± 0.08	0.9888	0.032
	SiO_2_-TRI-OXA	7.18 ± 0.46	0.9508	0.9803	1.002	0.60 ± 0.03	0.9857	0.072

**Table 2:** Thermodynamic parameters of the biomolecules’ adsorption processes on the dendrimer-grafted silica-supported materials.

Biomolecule	Adsorbent	Δ*H*° (kJ mol^−1^)	Δ*S*° (J mol^−1^ K^−1^)	R^2^	χ^2^·10^3^	Δ*G*° (kJ mol^−1^)		
						301 K	313 K	328 K
Folic Acid	SiO_2_-EDA	1.41 ± 0.12	43.8 ± 0.4	0.9855	0.002	−11.78	−12.32	−12.96
	SiO_2_-TETA	2.29 ± 0.02	51.8 ± 0.1	0.9998	0.001	−13.31	−13.93	−14.71
	SiO_2_-TREN	1.43 ± 0.09	44.2 ± 0.3	0.9924	0.001	−11.86	−12.39	−13.06
	SiO_2_-TRI-OXA	4.64 ± 0.28	53.3 ± 0.9	0.9929	0.007	−11.41	−12.03	−12.85
Salicylic Acid	SiO_2_-EDA	4.40 ± 0.13	47.3 ± 0.4	0.9982	0.641	−9.84	−10.42	−11.12
	SiO_2_-TETA	42.94 ± 3.02	147.6 ± 9.6	0.9901	4.545	−1.58	−3.12	−5.56
	SiO_2_-TREN	6.35 ± 0.21	53.7 ± 0.7	0.9979	0.649	−9.81	−10.43	−11.25
	SiO_2_-TRI-OXA	13.59 ± 0.76	60.0 ± 2.4	0.9938	0.157	−4.44	−5.22	−6.07
Nicotinic Acid	SiO_2_-EDA	4.38 ± 0.25	38.1 ± 0.8	0.9936	0.011	−7.10	−7.57	−8.13
	SiO_2_-TETA	9.42 ± 0.36	49.8 ± 1.1	0.9972	0.029	−5.59	−6.16	−6.94
	SiO_2_-TREN	6.26 ± 0.49	43.2 ± 1.6	0.9879	0.046	−6.72	−7.28	−7.89
	SiO_2_-TRI-OXA	10.45 ± 0.74	44.7 ± 2.4	0.9899	0.221	−3.04	−3.52	−4.25

**Table 3:** The drug release parameters calculated for the first-order, the Higuchi and the Korsmeyer-Peppas release models, based on the drug desorption experiments at pH 2.0.

Biomolecule	Adsorbent	First-Order Model		Higuchi Model		Korsemeyer-Peppas Model		
		k_1_ (% h^−1^)	R^2^ (χ^2^)	k_H_ (% h^−1/2^)	R^2^ (χ^2^)	n	k_K-P_ (% h^−1^)	R^2^ (χ^2^)
**Folic Acid**	SiO_2_-EDA	0.006 ± 0.002	0.5519 (0.039)	6.5 ± 1.8	0.7205 (10.340)	0.34	17.1 ± 1.8	0.8311 (0.064)
	SiO_2_-TETA	0.011 ± 0.004	0.5748 (0.116)	8.8 ± 2.4	0.7224 (10.763)	0.26	31.1 ± 2.1	0.8743 (0.020)
	SiO_2_-TREN	0.008 ± 0.003	0.5883 (0.058)	7.3 ± 1.9	0.7399 (9.740)	0.31	21.8 ± 2.0	0.8476 (0.041)
	SiO_2_-TRI-OXA	0.008 ± 0.003	0.6061 (0.061)	7.7 ± 1.9	0.7602 (10.207)	0.34	19.6 ± 1.9	0.8594 (0.048)
**Salicylic Acid**	SiO_2_-EDA	0.103 ± 0.030	0.7071 (7.928)	5.6 ± 2.3	0.5520 (3.596)	0.07	86.9 ± 2.0	0.8118 (0.002)
	SiO_2_-TETA	0.006 ± 0.003	0.5238 (0.038)	5.8 ± 1.7	0.6941 (6.777)	0.22	25.4 ± 1.5	0.8735 (0.015)
	SiO_2_-TREN	0.016 ± 0.008	0.4629 (0.319)	4.5 ± 1.8	0.5522 (2.629)	0.06	76.4 ± 1.6	0.8024 (0.002)
	SiO_2_-TRI-OXA	0.006 ± 0.003	0.4110 (0.036)	5.3 ± 2.0	0.5822 (7.903)	0.18	30.2 ± 1.9	0.7974 (0.018)
**Nicotinic Acid**	SiO_2_-EDA	0.029 ± 0.007	0.7707 (0.781)	7.6 ± 1.7	0.8062 (2.315)	0.10	70.5 ± 1.0	0.9613 (0.001)
	SiO_2_-TETA	0.007 ± 0.002	0.6211 (0.041)	6.5 ± 1.5	0.7847 (6.462)	0.29	19.1 ± 1.2	0.9061 (0.021)
	SiO_2_-TREN	0.028 ± 0.008	0.7378 (0.820)	5.2 ± 1.3	0.7596 (1.318)	0.06	79.8 ± 0.8	0.9443 (0.001)
	SiO_2_-TRI-OXA	0.020 ± 0.009	0.5047 (0.418)	12.7 ± 4.5	0.6164 (27.616)	0.31	40.1 ± 4.4	0.7941 (0.049)

## Supplementary Materials

The following are available online, Figure S1. The ESI-MS positive spectrum of ester intermediate, Figure S2. The ESI-MS positive spectra of the synthesized PAMAM dendrimers: (a) EDA, (b) TETA, (c) TREN and (d) TRI-OXA, Figure S3. The ESI-MS spectra (positive—top; negative—bottom) of exemplary TREN poly(amidoamine) dendrimer complexes with the studied biomolecules: (a) salicylic acid, (b) nicotinic acid, (c) folic acid, Figure S4. The Langmuir isotherm model fitted to the experimental data of the adsorption processes, Figure S5. The Freundlich isotherm model fitted to the experimental data of the adsorption processes, Figure S6. The Temkin isotherm model fitted to the experimental data of the adsorption processes, Figure S7. The Dubinin-Radushkevich isotherm model fitted to the experimental data of the adsorption processes, Figure S8. The thermodynamic plots of the biomolecules adsorption processes corresponding the van’t Hoff equation, Table S1. Fitting of the experimental data to the Tekmin and the Dubinin-Radushkevich isothermal models, Table S2. The drug release parameters calculated for the fitting of experimental data to the zero-order and the Hixson-Crowell release models.
