# Supplementary material for: Dendrimer-Functionalized Hybrid Materials Based on Silica as Novel Carriers of Bioactive Acids
Source: Molecules. 2020 Jun 8;25(11):2660. doi: 10.3390/molecules25112660 (PMC7321234; doi:10.3390/molecules25112660)
Supplement: Supplementary file 1 [file molecules-25-02660-s001.pdf]

Dendrimer-functionalized hybrid materials based on silica as novel carriers of bioactive acids

Mateusz Pawlaczyk [1,*], Grzegorz Schroeder [1]

[1] Faculty of Chemistry, Adam Mickiewicz University in Poznań, Uniwersytetu Poznańskiego 8, 61-614, Poznań, Poland

* Corresponding Author: mateusz.pawlaczyk@amu.edu.pl

Supplementary Information

Section A: The ESI-MS spectra of the synthesized dendrimers and their complexes with bioactive compounds studied

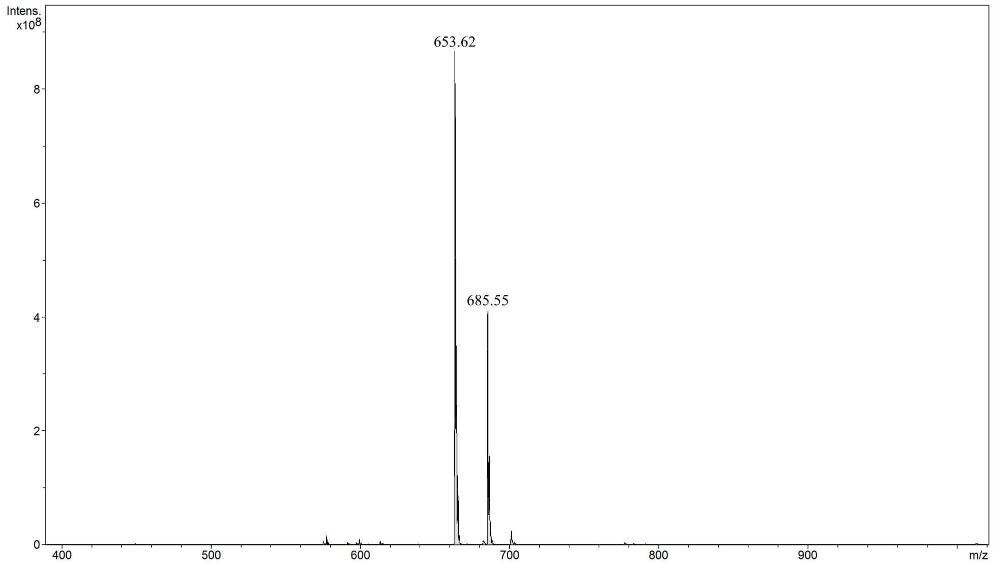

Figure S1. The ESI-MS positive spectrum of ester intermediate.

a)

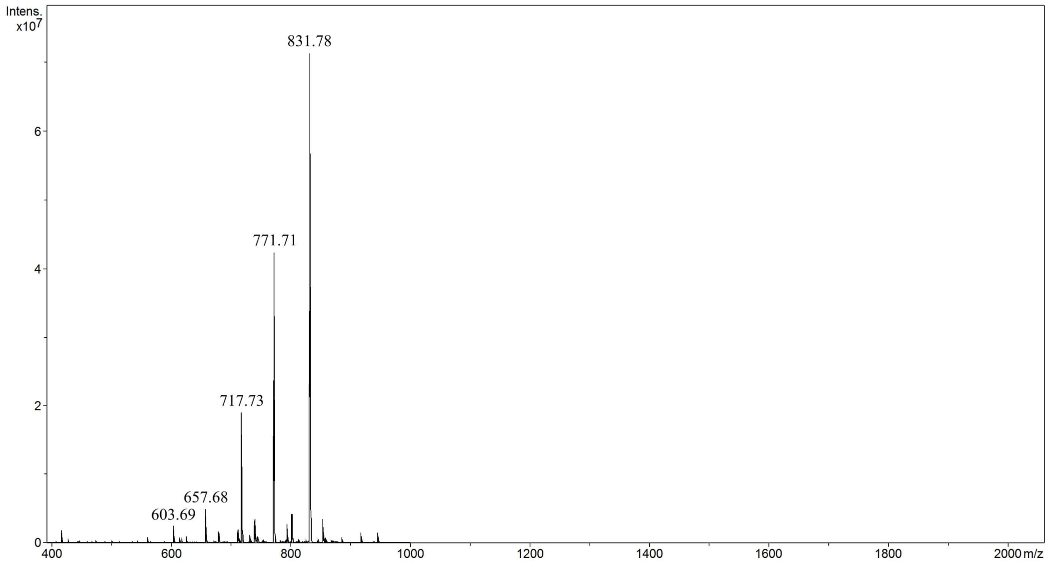

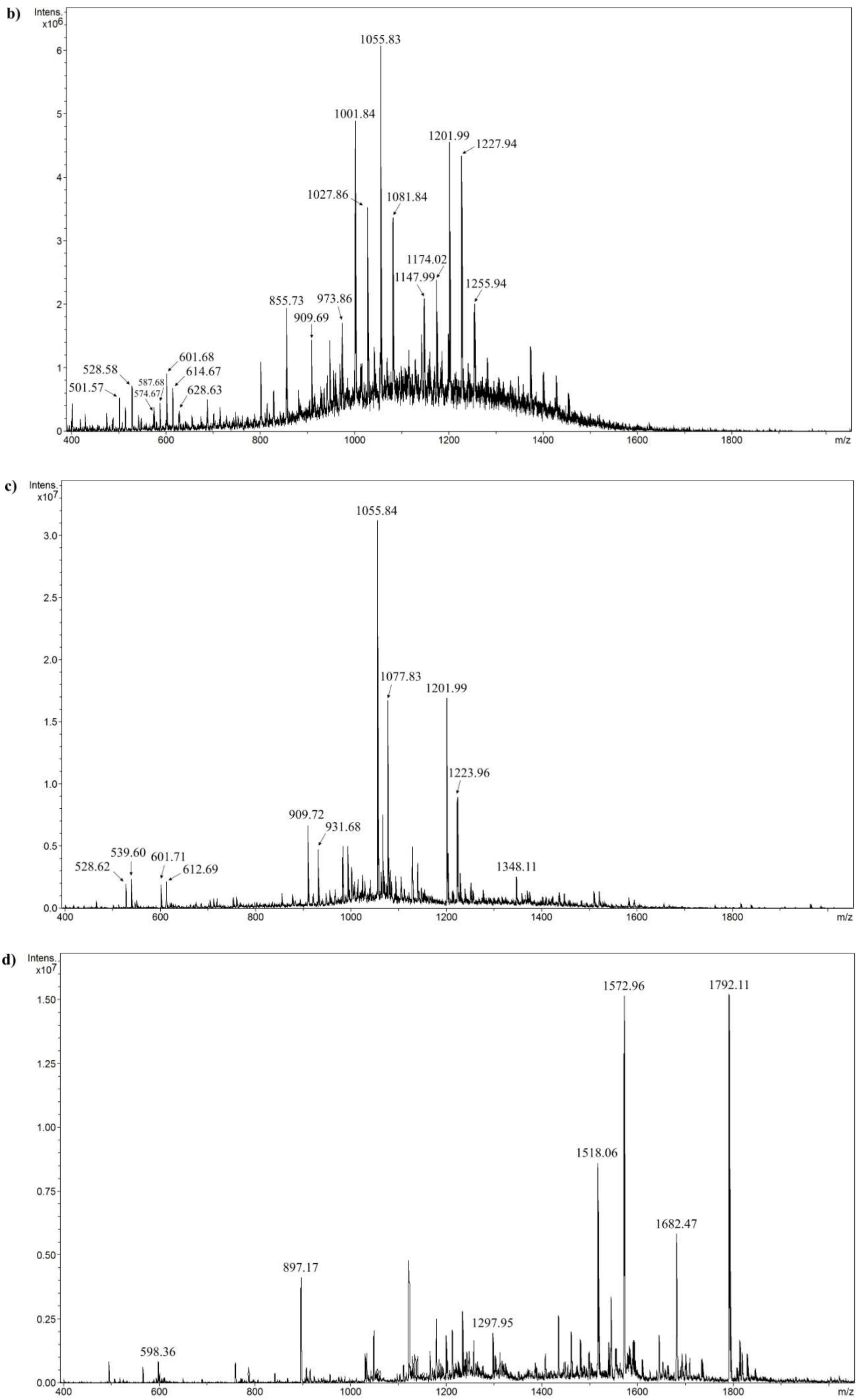

Figure S2. The ESI-MS positive spectra of the synthesized PAMAM dendrimers: (**a**) EDA, (**b**) TETA, (**c**) TREN and (**d**) TRI-OXA.

a)

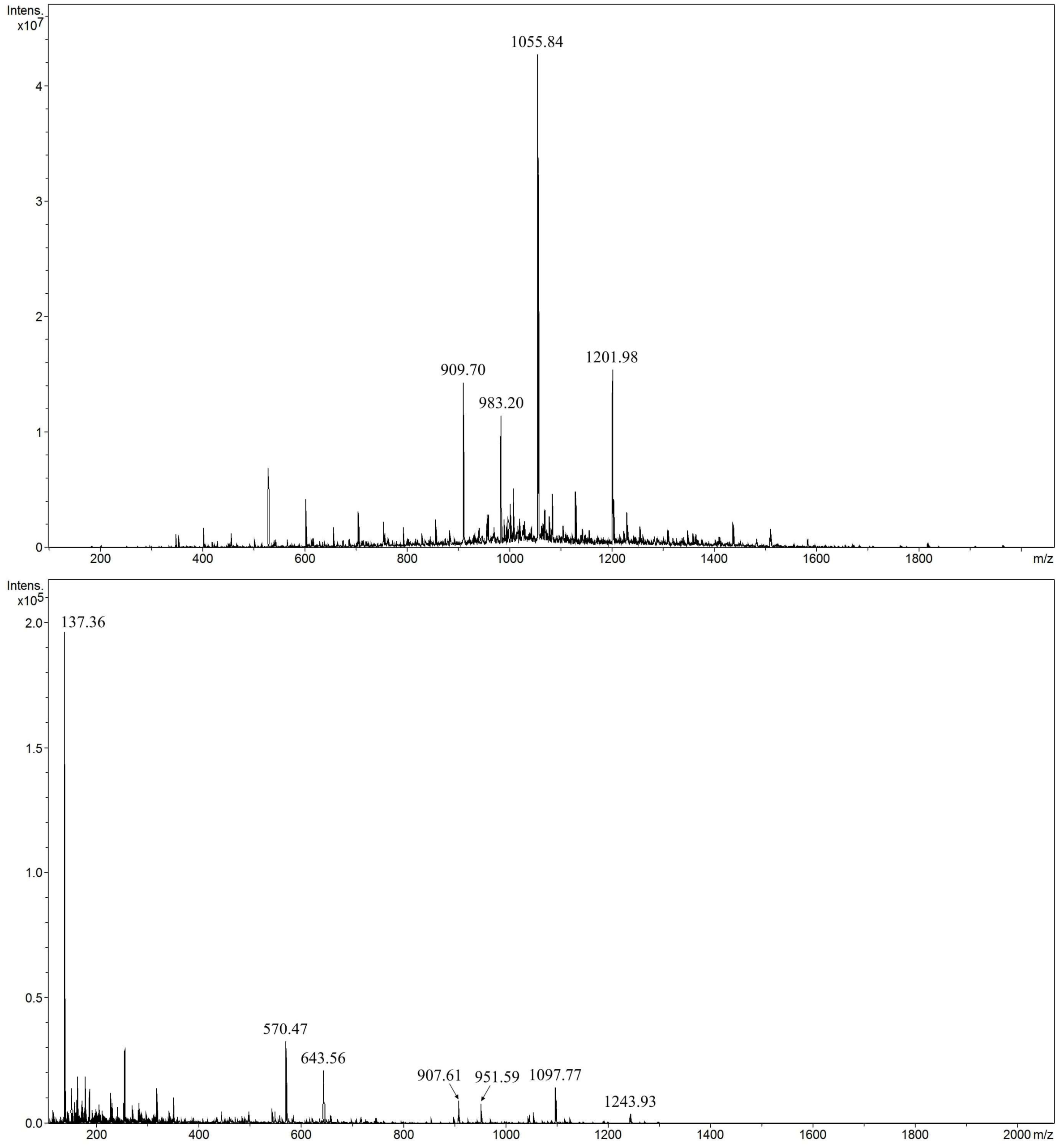
Intens.
x10^7
1055.84
909.70
983.20
1201.98
m/z
Intens.
x10^5
137.36
570.47
643.56
907.61
951.59
1097.77
1243.93
m/z

b)

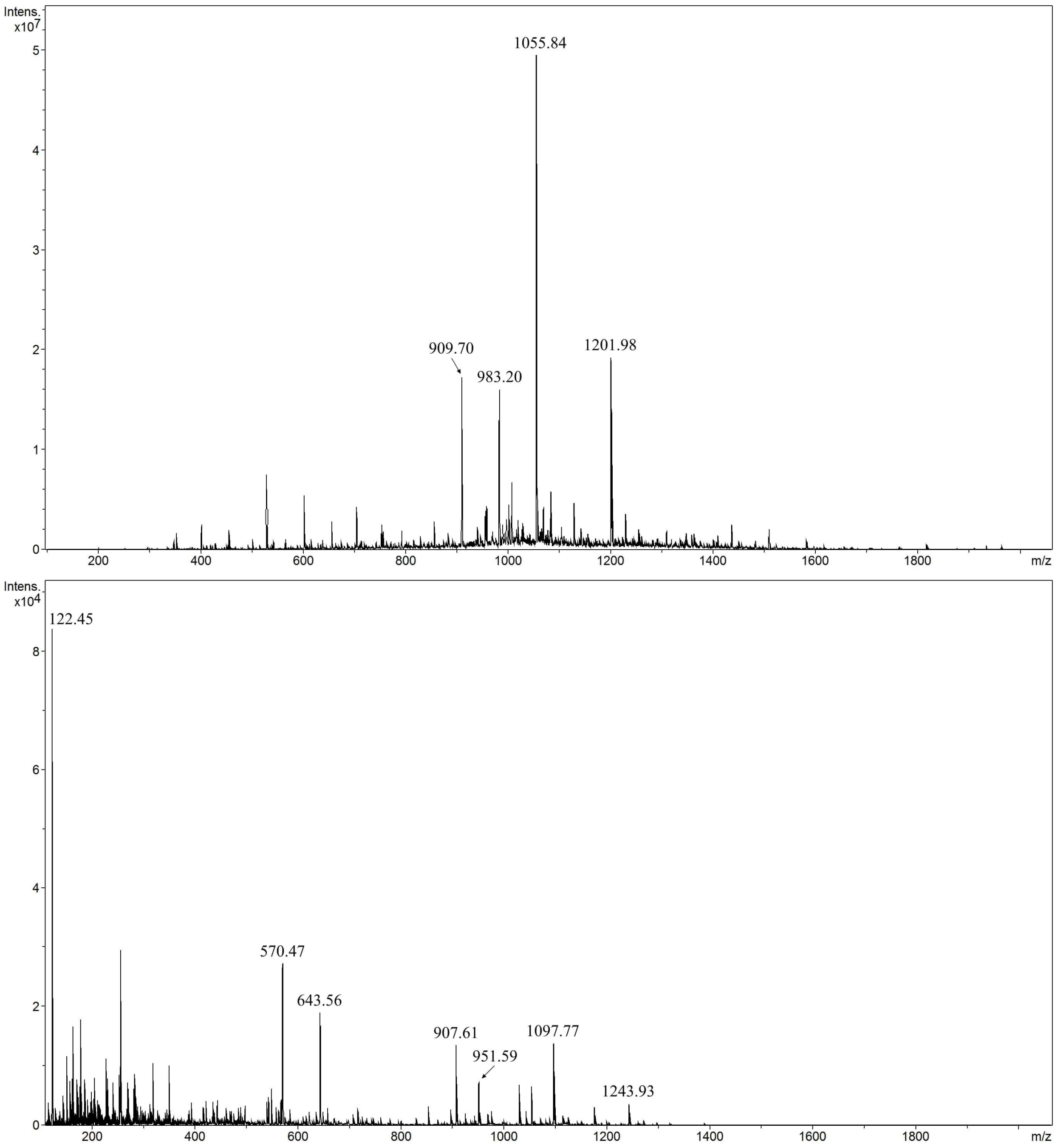
Intens.
x10^7
1055.84
909.70
983.20
1201.98
m/z
Intens.
x10^4
122.45
570.47
643.56
907.61
951.59
1097.77
1243.93
m/z

c)

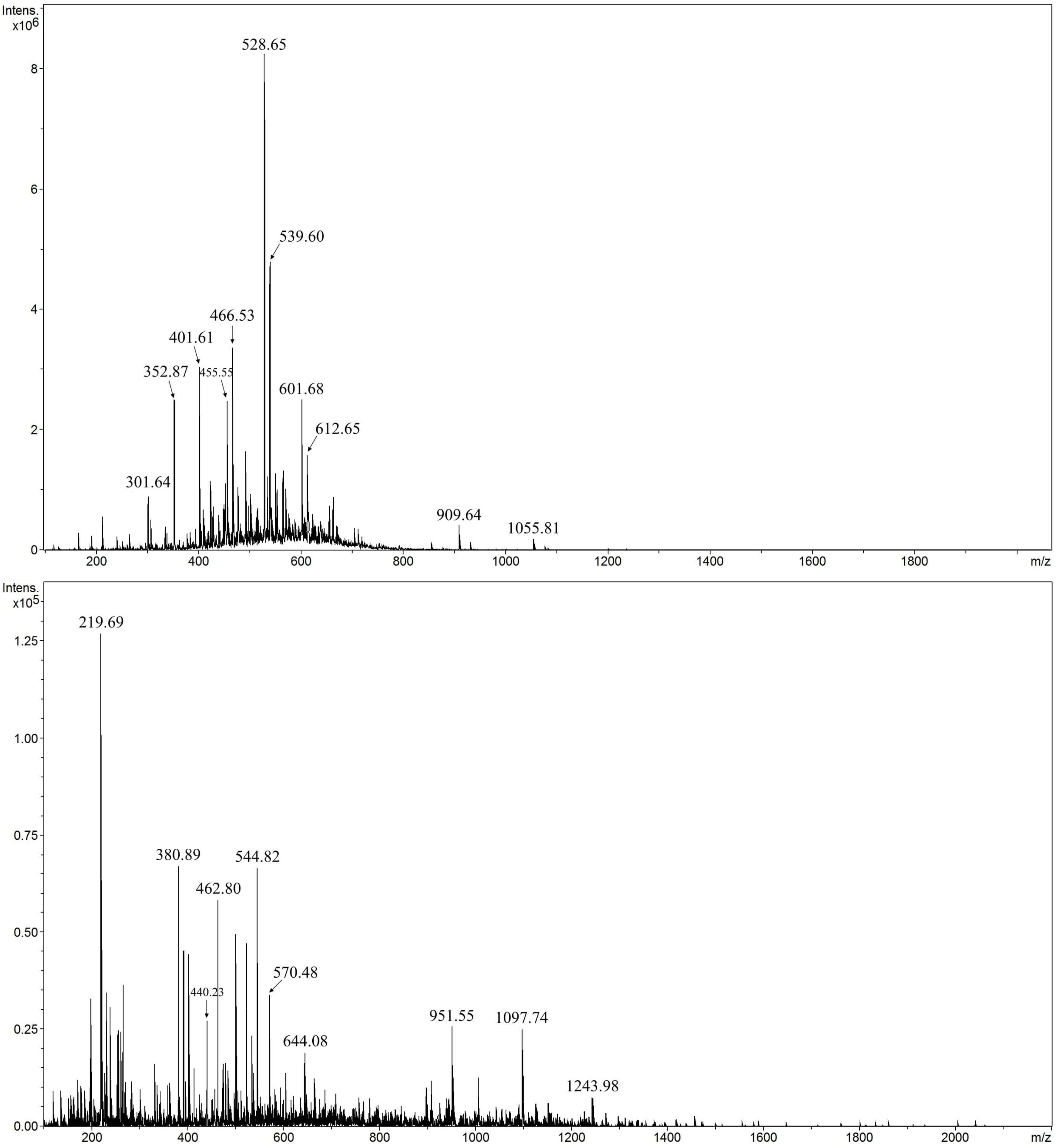

Figure S3. The ESI-MS spectra (positive – top; negative – bottom) of exemplary TREN poly(amidoamine) dendrimer complexes with the studied biomolecules: (**a**) salicylic acid, (**b**) nicotinic acid, (**c**) folic acid.

Section B: The supplement of the conducted adsorption experiments

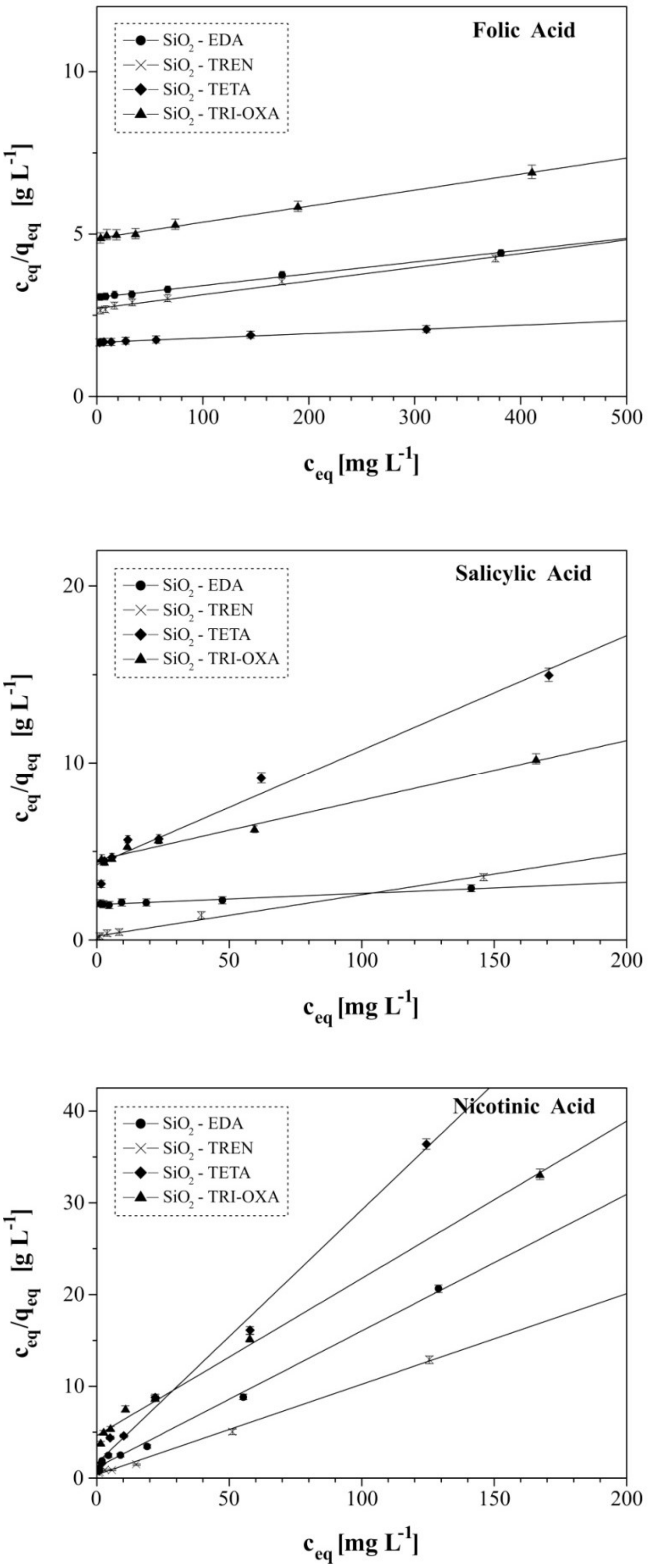

Figure S4. The Langmuir isotherm model fitted to the experimental data of the adsorption processes. For some points SDs are smaller than the plotted symbols.

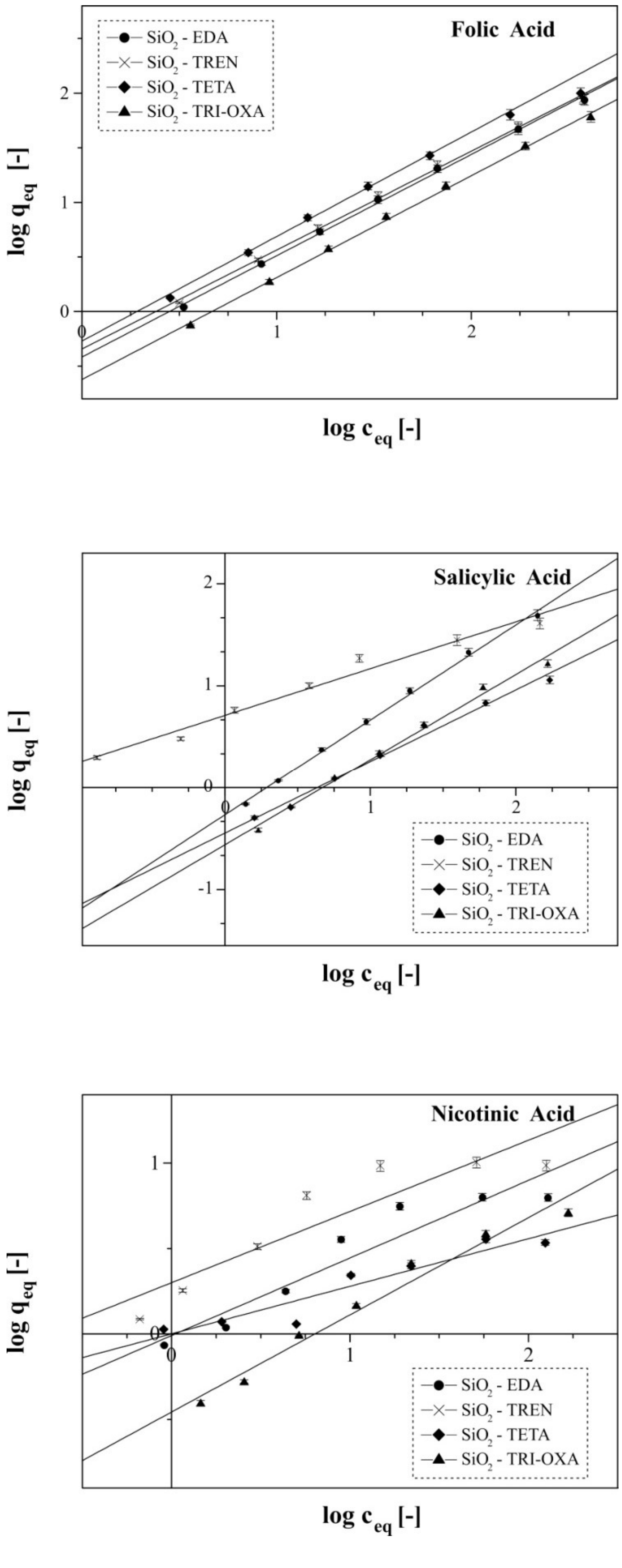

Figure S5. The Freundlich isotherm model fitted to the experimental data of the adsorption processes. For some points SDs are smaller than the plotted symbols.

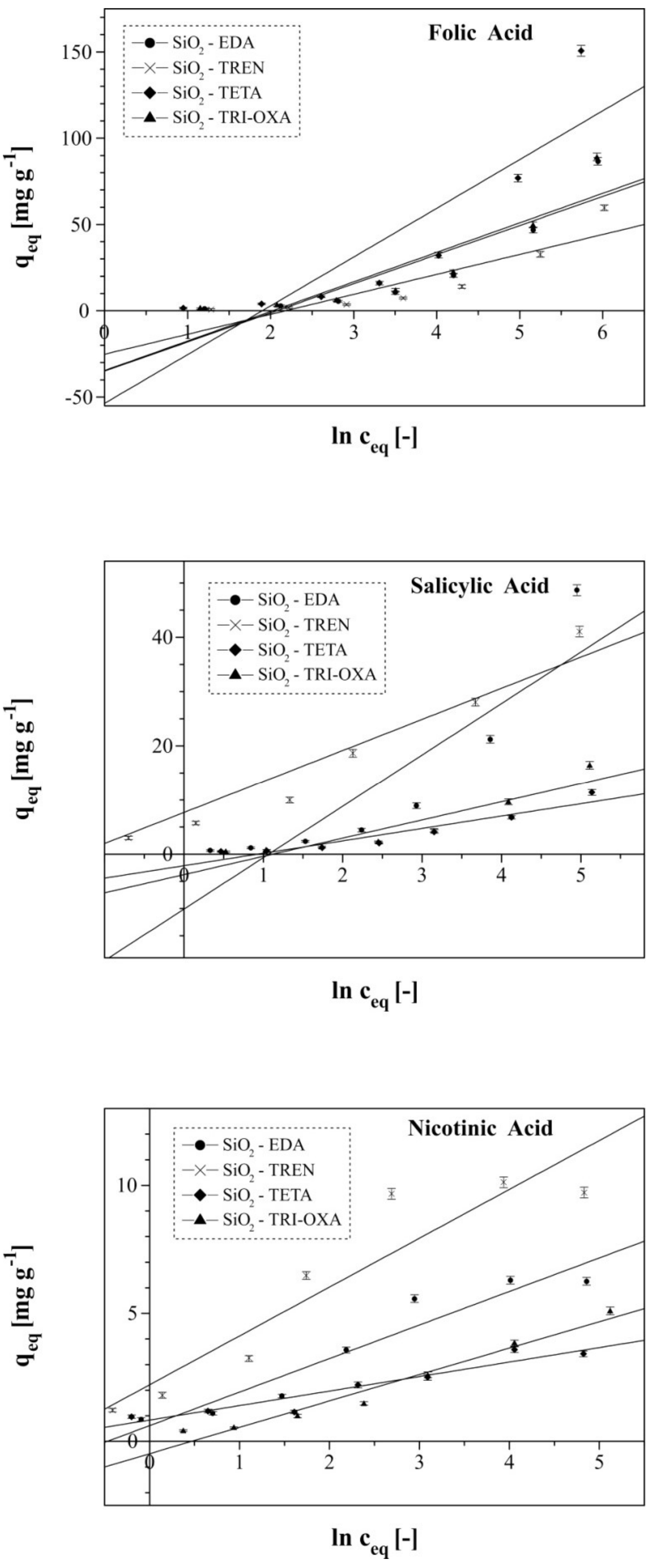

Figure S6. The Temkin isotherm model fitted to the experimental data of the adsorption processes. For some points SDs are smaller than the plotted symbols.

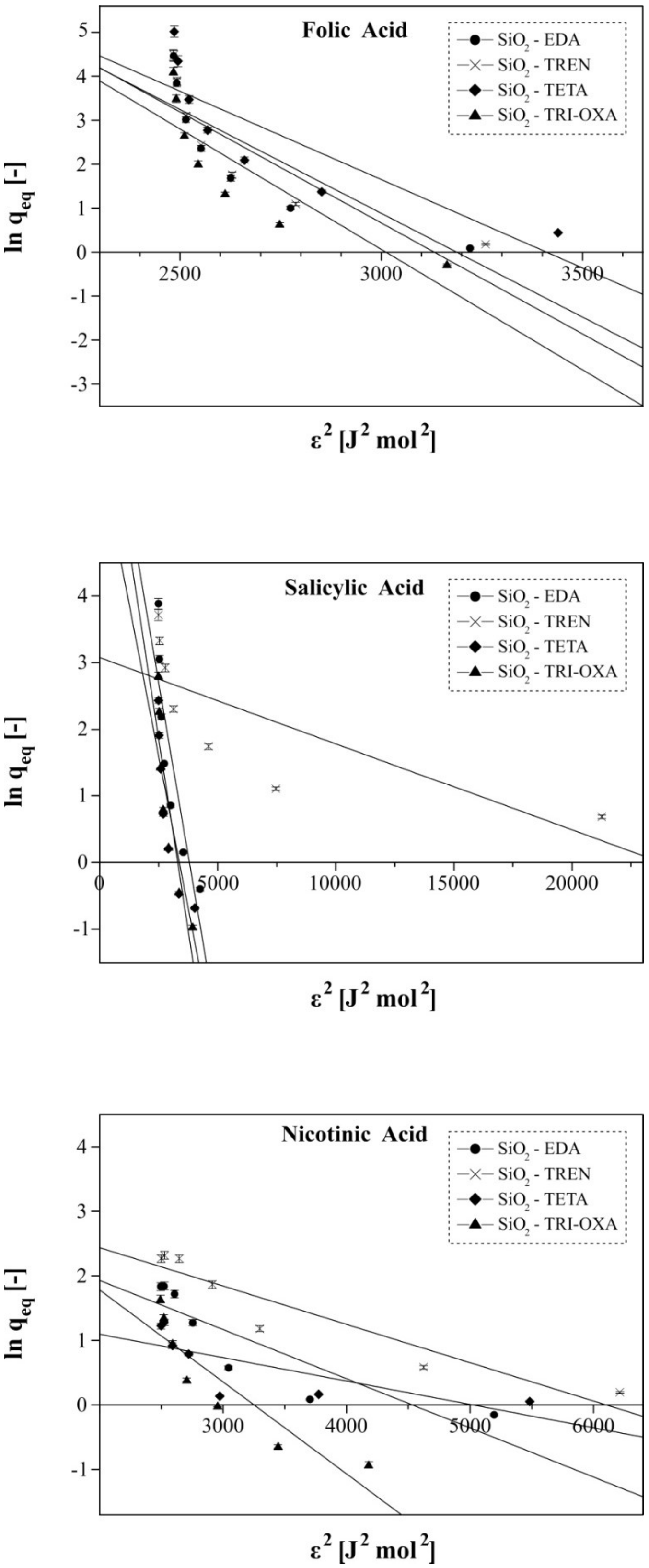

Figure S7. The Dubinin-Radushkevich isotherm model fitted to the experimental data of the adsorption processes. For some points SDs are smaller than the plotted symbols.

Table S1. Fitting of the experimental data to the Tekmin and the Dubinin-Radushkevich isothermal models.

Biomolecule	Adsorbent	Temkin Isotherm			Dubinin-Raduschkevich Isotherm		
		B [J mol^{-1}]	R^2	χ^2	E [kJ mol^{-1}]	R^2	χ^2
Folic Acid	SiO_2-epoxy	1.54	0.8608	0.010	0.209 ± 0.075	0.6052	43.592
	SiO_2-EDA	16.75	0.7564	16.250	0.260 ± 0.106	0.5474	9.514
	SiO_2-TETA	19.83	0.8038	7.689	0.296 ± 0.115	0.5717	9.091
	SiO_2-TREN	17.07	0.7635	12.862	0.273 ± 0.110	0.5508	9.160
	SiO_2-TRI-OXA	11.58	0.7532	9.980	0.242 ± 0.099	0.5440	10.392
Salicylic Acid	SiO_2-epoxy	0.25	0.9490	0.512	0.406 ± 0.108	0.7376	6.140
	SiO_2-EDA	9.47	0.7628	27.602	0.526 ± 0.185	0.6173	12.798
	SiO_2-TETA	3.31	0.8354	8.626	0.462 ± 0.156	0.6366	14.136
	SiO_2-TREN	5.73	0.9098	12.498	2.448 ± 0.927	0.5824	6.369
	SiO_2-TRI-OXA	2.82	0.8713	19.115	0.496 ± 0.166	0.6426	12.631
Nicotinic Acid	SiO_2-epoxy	0.22	0.9299	0.636	0.020 ± 0.005	0.7540	61.718
	SiO_2-EDA	2.99	0.8557	6.049	0.871 ± 0.373	0.5207	8.278
	SiO_2-TETA	0.84	0.9587	0.473	0.813 ± 0.257	0.6676	6.779
	SiO_2-TREN	2.90	0.9294	6.128	0.997 ± 0.314	0.6593	7.170
	SiO_2-TRI-OXA	1.18	0.9221	2.068	0.620 ± 0.219	0.6359	11.258

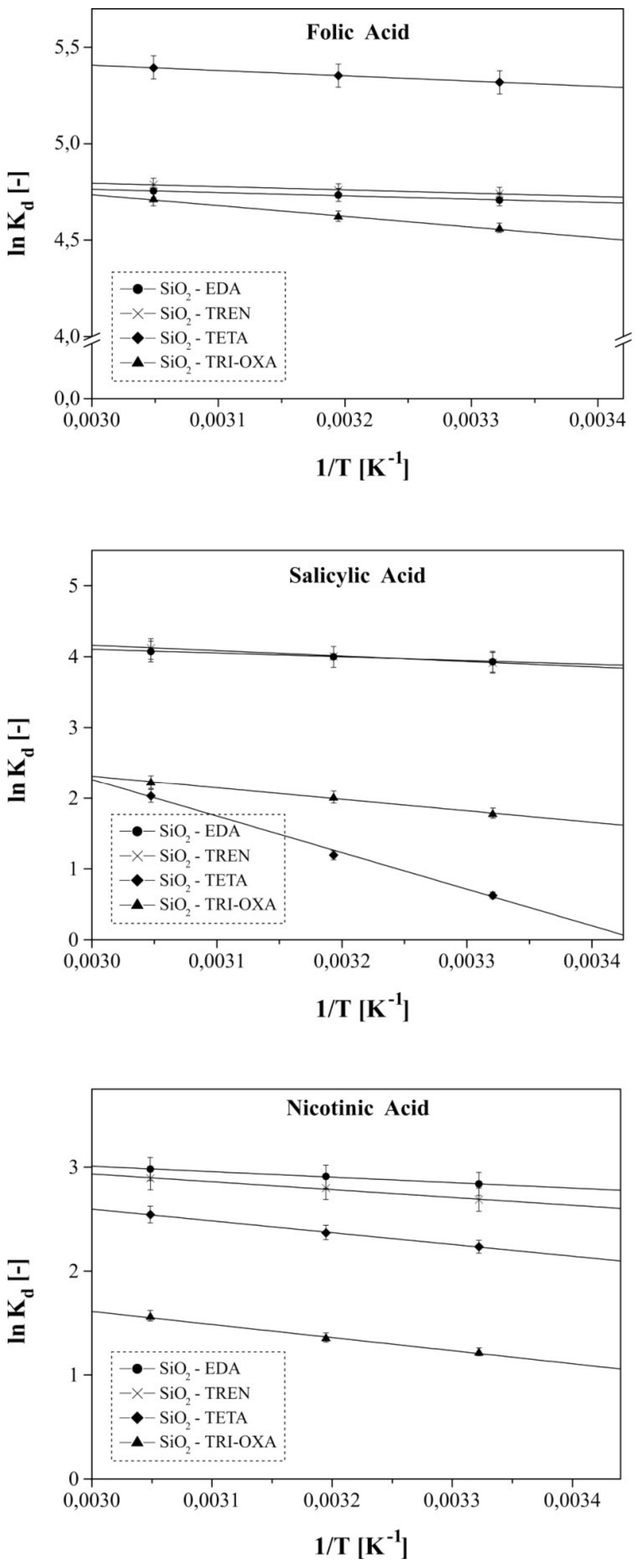

Figure S8. The thermodynamic plots of the biomolecules adsorption processes corresponding the van't Hoff equation. For some points SDs are smaller than the plotted symbols.

Section C: The supplement of the conducted drug-release experiments

Table S2. The drug release parameters calculated for the fitting of experimental data to the zero-order and the Hixson-Crowell release models.

**Biomolecule**	**Adsorbent**	**Zero-Order Model**		**Hixson-Crowell Model**	
		**k_1 [mg h^{-1}]**	**R^2 (χ^2)**	**k_{H-C} [$mg^{1/3}$ h^{-1}]**	**R^2 (χ^2)**
Folic Acid	SiO_2-EDA	0.006 ± 0.002	0.5433 (0.085)	0.008 ± 0.004	0.4265 (0.078)
	SiO_2-TETA	0.005 ± 0.002	0.5477 (0.058)	0.007 ± 0.003	0.4596 (0.073)
	SiO_2-TREN	0.005 ± 0.002	0.5698 (0.061)	0.007 ± 0.004	0.4599 (0.063)
	SiO_2-TRI-OXA	0.004 ± 0.001	0.5860 (0.059)	0.008 ± 0.004	0.4649 (0.072)
Salicylic Acid	SiO_2-EDA	0.007 ± 0.004	0.3547 (0.042)	0.003 ± 0.002	0.3371 (0.111)
	SiO_2-TETA	0.006 ± 0.002	0.4885 (0.066)	0.006 ± 0.003	0.4280 (0.039)
	SiO_2-TREN	0.006 ± 0.004	0.3611 (0.032)	0.003 ± 0.002	0.3416 (0.071)
	SiO_2-TRI-OXA	0.005 ± 0.003	0.3809 (0.069)	0.005 ± 0.003	0.3374 (0.056)
Nicotinic Acid	SiO_2-EDA	0.004 ± 0.001	0.6156 (0.013)	0.004 ± 0.001	0.5821 (0.043)
	SiO_2-TETA	0.004 ± 0.001	0.5863 (0.045)	0.006 ± 0.002	0.5729 (0.032)
	SiO_2-TREN	0.004 ± 0.002	0.5655 (0.011)	0.001 ± 0.002	0.0592 (0.156)
	SiO_2-TRI-OXA	0.008 ± 0.004	0.4099 (0.159)	0.008 ± 0.005	0.3438 (0.235)
